# Incoherent feedforward loop dominates the robustness and tunability of necroptosis biphasic, emergent, and coexistent dynamics

**DOI:** 10.1016/j.fmre.2024.02.009

**Published:** 2024-03-05

**Authors:** Fei Xu, Xiang Li, Rui Wu, Hong Qi, Jun Jin, Zhilong Liu, Yuning Wu, Hai Lin, Chuansheng Shen, Jianwei Shuai

**Affiliations:** a Department of Physics and Fujian Provincial Key Laboratory for Soft Functional Materials Research, Xiamen University, Xiamen 361005, China; b National Institute for Data Science in Health and Medicine and State Key Laboratory of Cellular Stress Biology, Innovation Center for Cell Signaling Network, School of Life Sciences, Xiamen University, Xiamen 361102, China; c Complex Systems Research Center, Shanxi University, Taiyuan 030006, China; d Department of Mathematics and Physics, Fujian Jiangxia University, Fuzhou 350108, China; e Oujiang Laboratory (Zhejiang Lab for Regenerative Medicine, Vision and Brain Health) and Wenzhou Institute, University of Chinese Academy of Sciences, Wenzhou 325001, China; f School of Mathematics and Physics, Anqing Normal University, Anqing 246011, China; g Department of Physics, Anhui Normal University, Wuhu 241002, China

**Keywords:** Incoherent feedforward loop, Biphasic dynamics, Emergence, Necroptosis, Design principle

## Abstract

Biphasic dynamics, the variable-dependent ability to enhance or restrain biological function, is prevalent in natural systems. Accompanied by biphasic dynamics, necroptosis signaling also appears emergent and coexistent dynamics. However, it remains elusive how the properties of these dynamics are characterized by specific circuit structures and components. Starting with necroptosis circuit modeling, we systematically analyzed the network topology for achieving RIP1-dependent biphasic, emergent, and coexistent (BEC) dynamics. RIP1-RIP3-Caspase-8 (C8) incoherent feedforward loop embedded with positive feedback of RIP3 to RIP1 is identified as the core topology. The peak value of RIP3 phosphorylation is determined to present a scale-invariant feature, dictating BEC dynamics and the bell-shaped regulation of necroptosis biphasic dynamics. To quantitatively determine the uncertainty of necroptosis coexistent dynamics, potential landscape and Shannon entropy that measure entropy production during cell death are introduced for the first time. Further random necroptosis circuit analysis identifies the bell-shaped regulation of necroptosis biphasic dynamics by RIP3 auto-phosphorylation, which acts as a complementary process for robustly attaining BEC dynamics. Finally, we searched all possible two- and three-node circuit topologies to screen those that could perform BEC dynamics. A complete atlas of three-node circuit BEC dynamics is generated and only three minimal circuits emerge as robust solutions, confirming incoherent feedforward loop is the core topology. Analysis of the association between the minimal circuit structure and robustness proves that the identified optimal functional achievement structure is highly consistent with the experimental observed RIP1-RIP3-C8 topology. Overall, through highlighting a finite set of circuits, this study yields guiding principles that enable the mapping, modulation, and design of circuits for BEC dynamics in diverse synthetic biology applications.

## Graphical abstract

**Figure:**
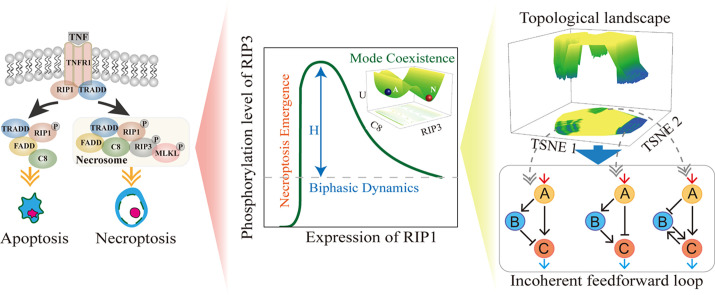

## 1 Introduction

Biphasic behavior is a common phenomenon observed in a wide range of biological processes that are essential for physiological and developmental functions, such as cell differentiation [1], proliferation [2], and death [3]. Biphasic behavior can be categorized as either time-dependent or dose-dependent [4]. Time-dependent biphasic behavior refers to a response that initially increases with time but eventually decreases, or vice versa. Examples of time-dependent biphasic behavior include pulse and adaptation, such as the transient ERK activation induced by growth factors [5] and the PhoQ activation induced by ADP affinity in bacterial two-component systems [6]. Dose-dependent biphasic behavior refers to a response that initially increases or decreases with input, then eventually decreases or increases. Examples of this type of behavior include the biphasic dose dependence on Norepinephrine in cyclic AMP signaling [7], and the blue-light-dependent phosphorylation of Arabidopsis cryptochrome 2 in HEK293 cells [8].

Biphasic behavior in biological systems often results in the emergence of new patterns when it crosses a tipping point. Emergent dynamics are the biological function outcomes of collective interactions among various components, such as the patterns of chimera states and synchronization triggered by cell-to-cell interactions [9]. As a key upstream regulator, the role of RIP1 in dictating cell death is perplexing [10]. Recently, we found RIP1 biphasically regulates RIP3 phosphorylation with necroptosis emergence in TNF-induced cell death signaling [11]. However, the intrinsic relationship between signaling topology and RIP3 biphasic dynamics with emergence, and how the biphasic dynamics are regulated, have not been elucidated. Exploring the link between biological functions and the design principles of biological networks is a fundamental challenge to understand how living organisms can perform various functions efficiently and accurately. The theories of network science have been proven to be powerful tools [12]. Despite the apparent complexity and diversity of cell signaling, only a limited number of topologies might be capable of robustly executing particular function. Ma et al. successfully dissected the principle for the design of network topologies that robustly achieve adaptation [13]. Li et al. validated that the robustness of biological oscillators is enhanced by the incoherent inputs [14]. The design principle for robust oscillatory behaviors with respect to noise has also been demonstrated recently [15]. This study aims to demonstrate the general rules of necroptosis signaling that achieve biphasic, emergent, and coexistent (BEC) dynamics, and seeks to explore the design principles present in natural systems. This will help to better understand how living organisms can perform these dynamics efficiently and accurately.

## 2 Materials and methods

### 2.1 Cell line and cell culture

Mouse fibrosarcoma L929 were obtained from ATCC. RIP1 KO, RIP3 KO, L929, TRADD KO and Caspase-8 (C8) KO L929 cells were generated by TALEN or CRISPR/Cas9 methods. The knock-out cells were determined by sequencing of targeted loci and immunoblotting of the expression of respective proteins. All cells were maintained in Dulbecco’s modified Eagle’s medium (DMEM), supplemented with 10% fetal bovine serum, 2 mM l-glutamine, 100 IU penicillin, and 100 mg/ml streptomycin at 37 °C in a humidified incubator containing 5% CO_2_. The target sites were designed as follows: RIP3: “CTAACATTCTGCTGGA”; RIP1: “AACCGCGCTGAGTGAGTTGG”; TRADD: “AAGATGGCAGCCGGTCAGAA”; Caspase-8: “GTGTTCAAATACATACGCCT”. All lentiviral-shRNAs were constructed into pLV-H1-EF1α-puro vector or pLV-H1TetO-GFP-Bsd following the manufacturer's instruction (Biosettia). The indicated shRNA target sequence was: RIP1 shRNA: 5′-GCATTGTCCTTTGGGCAAT-3′.

### 2.2 Reagents and antibodies

Mouse TNF-α were obtained from eBioscience (San Diego, CA, USA). Anti-RIP3 (dilution 1:1,000) and anti-MLKL (dilution 1:1,000) were raised using E. coli-expressed GST-RIP3 (287–387 amino acid), GST-MLKL (100–200 amino acids) and GST-FADD (full length), respectively. Anti-caspase-8 antibody (4,790, dilution 1:1,000) and anti-cleaved caspase-8 antibody (8,592, dilution 1:500) were purchased from Cell Signaling Technology. Anti-p-RIP3 antibody (ab222320, dilution 1:500) and anti-p-MLKL antibody (ab196436, dilution 1:1,000) were purchased from Abcam. Anti-Gapdh antibody (60,004–1-Ig, dilution 1:2,000) was from Proteintech. Anti-RIP1 antibody (610,459, dilution 1:1,000) was from BD Biosciences.

### 2.3 Immunoprecipitation and western blotting

Cells were seeded in a 100 mm dish, grew to reach confluency. After stimulating, cells were washed by PBS for three times and then lysed with lysis buffer (20 mM Tris–HCl, pH 7.5, 150 mM NaCl, 1 mM Na2EDTA, 1 mM EGTA, 1% Triton X-100, 2.5 mM sodium pyrophosphate, 1 mM β-glycerophosphate, 1 mM Na3VO4) on ice for 30 min. Cell lysates were then centrifuged at 20,000 g for 30 min. The supernatant was immunoprecipitated with anti-Flag M2 beads at 4 °C overnight. After the immunoprecipitation, the beads were washed three times in lysis buffer and the immunoprecipitated proteins were subsequently eluted by SDS sample buffer with 0.15 µg/µL 3× Flag peptide.

### 2.4 Microscopy imaging of cell death

To examine cell death morphology, cells were treated as indicated in 12-well plates or 35-mm glass bottom dishes for image capture. Static bright-field images of cells were captured using Zeiss LSM 780 at room temperature. The pictures were processed using Image J or the ZEN 2012 Image program.

### 2.5 Necroptosis circuit modeling

TNF-mediated cell death circuit can be mathematically modeled using the law of mass action and simple assumptions [14]. For proteins A and B, a simple process of A activating B is described as follows:

(1) $$B+nA\underset{k_{off}}{\overset{k_{on}}{⇌}}BA_{n}\overset{k}{\to }B^{*}+nA$$

At steady-state:

(2) $$d\left[BA_{n}\right]/dt=k_{on}\left[B\right]{\left[A\right]}^{n}-\left(k_{off}+k\right)\left[BA_{n}\right]=0$$

Assuming the binding between proteins is independent, and the dissociation rate of the complex is much larger than the binding rate.

(3) $$\left[BA_{n}\right]={\left[B\right]}_{tot}-{\left[B\right]}^{*}-\left[B\right]$$

With the normalized total amount of proteins, the rate of A mediated activation of B can be described as follows:

(4) $$v=k\left(1-{\left[B\right]}^{*}\right)\frac{{\left[A\right]}^{n}}{{\left[A\right]}^{n}+\left(k_{off}+k\right)/k_{on}}$$

### 2.6 Noise and potential landscape

There is randomness in the procedure of biochemical reactions in cells, including intrinsic randomness, that is, molecular noise, and thermal fluctuations in the biochemical environment [[16], [17]]. For simplicity, we add a noise term, *σdξ*, to the OEDs of the deterministic model and assume that the noise intensity is correlated with the protein level. *ξ* represents for white Gaussian noise, and the statistical properties satisfy < *ξ_i_*(*t*) > = 0 and < *ξ_i_*(*t*)*ξ_i_*(*t’*) > = 2*σδ* (*t*-*t’*).

The global dynamics of a stochastic system with noise are given by the potential landscape. The stochastic dynamics of cell death fate decision system could be characterized by the generalized Langevin equation *dx_i_*(*t*)/*dt* = ***F***(*x_i_*) + *ξ*(*t*), where *x* represents the concentrations of the proteins and ***F*** is the driving force. The Fokker-Planck equation describes the evolution of the probability density p in the state space, as follows: ∂*p*(*x_i_, t*)/∂*t* = -Σ∂(*F*(*x_i_*) *p*(*x_i_, t*))/∂*x_i_* + *D_i_*Σ∂^2^*p*(*x_i_, t*)/∂*x_i_*^2^. Since the dimensionality of the model limits the direct access to the probability density through the evolution of the Fokker–Planck equation, we use the Bernoulli experiment numerical method to replace the steady-state probability distribution with the trajectory density distribution in the phase space. Specifically, we divide the two-dimensional phase space of RIP3 and C8 into 200 × 200 lattices and assign 10,000 sets of random initial conditions to the stochastic differential equations. After a long enough evolution, 10,000 trajectories can be obtained in the phase space, and the number of trajectories in each lattice is counted and the density is calculated [18].

### 2.7 Parameter selection and sampling

In our study, the parameter ranges of the computational models were consistent with those in previous publications of similar studies [13,19,20]. For each topology, 50,000 parameter sets are uniformly sampled using Latin hypercube sampling, with parameters ranging from *k*∼0.1–10 (logarithmic scale), *j*∼0.001–100 (logarithmic scale), *d*∼0.01–1 (logarithmic scale), *n*∼1–4 (integer scale), stimulation signal∼0–1.

## 3 Results

### 3.1 Necroptosis BEC dynamics within TNF-induced cell death circuit

**Fig. 1:**
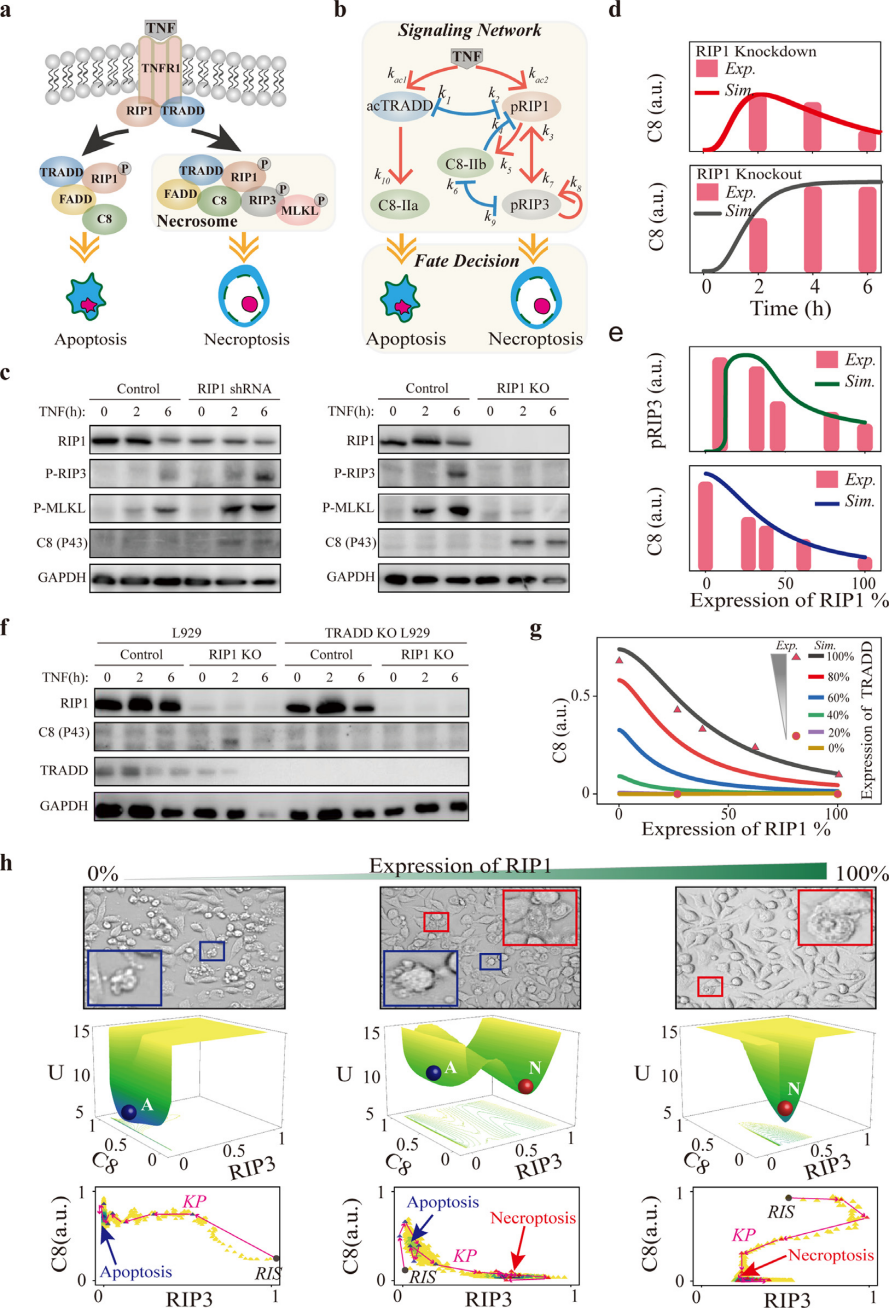
**Data-driven modeling of the TNF-induced cell death circuit.** (a) Schematic diagram of TNF-induced apoptosis and necroptosis signaling pathway. (b) The coarse-grained signaling network model. (c) Western blot analysis of the effects of RIP1 knockdown (shRNA) or knockout (KO) on indicated proteins activation. (d) Comparison between experimental data (histograms) and simulation results (lines) of the time-course responses of C8 in RIP1 knockdown (upper panel) and RIP1 knockout (down panel) cells. (e) Comparison between experimental data (histograms) and simulation results (lines) of RIP1-dependent pRIP3 response (upper panel) and C8 activation response (down panel). (f) Western blot analysis of TRADD knockout on indicated protein activation for wildtype and RIP1 knockout cells. (g) Comparison between experimental data (dots) and simulation results (lines) of the effect of TRADD level on RIP1-dependent C8 activation. (h) Cell morphologies under different expression levels of RIP1. The red and blue boxes indicate the represented apoptotic and necroptotic cells, respectively. Potential landscape reveals switch in cell death modes under different levels of RIP1 and the corresponding kinetic pathways (KP) of system evolution from random initial states (RIS). Colored dots indicate instantaneous positions, with color reflecting dot density.

TNF is a multi-functional cytokine that can induce apoptosis or necroptosis depending on cellular contexts [21]. The schematic diagram of TNF-induced apoptosis and necroptosis signaling pathway is shown in Fig. 1a. To intuitively address the relation of the core module, the reactions, such as association/disassociation, and cascades reaction can be coarsely described to present a conceptual core signaling circuit as shown in Fig. 1b. Upon stimulation, TNF combines with TNFR1 to recruit TRADD and RIP1 to form complex-I and then activates them (Fig. 1a) [22], which can be simplified as TNF activating TRADD and RIP1 in Fig. 1b (Table 1). The competition between TRADD and RIP1 for binding TNFR1 is described as mutual inhibition [23]. Activation of caspase-8 (C8) by sufficient TRADD in complex-II (labeled as C8-IIa) could result in apoptosis. When apoptosis occurs, the cell volume becomes small and the nucleus shrinks [24]. Besides, C8 could also be activated by RIP1 in necrosome (labeled as C8-IIb) [25]. In necrosome, C8 inhibits the phosphorylation of RIP1 and RIP3 through cleaving RIP1-RIP3 complex [26]. Phosphorylation of RIP3, the marker of necroptosis [21], also blocks C8 activation by recruiting RSK [27]. RIP1 and RIP3 activate each other through their RHIM-domain, forming a positive feedback loop for the recruitment of MLKL and necroptosis induction [28]. As a result, the TNF-induced death dynamics are mainly determined by five components, i.e., activated TRADD (acTRADD), phosphorylated RIP1 (pRIP1), phosphorylated RIP3 (pRIP3), C8 activated by TRADD (C8-IIa), and C8 activated by RIP1 (C8-IIb) (Fig. 1b).

As shown by the western blotting data in Fig. 1c, RIP1 knockdown (RIP1 shRNA) accelerates RIP3/MLKL phosphorylation, while RIP1 deletion (RIP1 KO) completely blocks RIP3/MLKL phosphorylation, presenting a biphasic dynamic of necroptosis regulated by RIP1. Decrease of RIP1 also promotes C8 activation, suggesting the suppression role of RIP1 in apoptosis (Fig. 1c). In line with prior studies [11], the quantification of the data depicted in Fig. 1c indicates a gradual increase in both the levels of pRIP3 and pMLKL following TNF stimulation (Fig. S1). To fully understand the essential topology for achieving the biphasic dynamics in the death signaling, a self-evolving ODEs model is constructed based on the circuit shown in Fig. 1b. MLKL serves as a downstream substrate of RIP3, presenting similar dynamics to RIP3 and acting as an executor of necroptosis (Fig. 1c). Besides, previous studies indicated that RNA interference-mediated downregulation of MLKL does not impact the upstream signaling of RIP1 and RIP3 [29]. Therefore, for simplification in our coarse-grained model, we only considered RIP3 dynamics. Recently, Xiong and his colleagues identified VDAC1 as acting downstream of MLKL [30], which could be considered in our future work. The circuit model well reproduces the experimental observations of C8 and RIP3 activation under different RIP1 levels (Fig. 1d, 1e). pRIP3 presents an abrupt and large increase at low level of RIP1, triggering the emergent dynamics of necroptosis (Fig. 1e). pRIP3 then gradually decreases with further increases in RIP1, while C8 activation exhibits a linear reduction with increasing RIP1. Experiments show that the deletion of RIP1-induced C8 activation is completely blocked in TRADD deletion cells (Fig. 1f), proving that RIP1 suppresses apoptosis through restraining TRADD-dependent C8 activation. Consistently, our circuit model can also quantitatively reproduce the experimental observations and further provides a comprehensive analysis result, showing the decrease of TRADD results in a progressive reduction of C8 activation at varying RIP1 levels (Fig. 1g). Previous study demonstrated that L929 cells exclusively undergo necroptosis upon TNF stimulation, and the death rate increases with the intensity of TNF stimulation [31]. Besides, potential of TNF-induced necroptosis as a consequence of RIP1 knockdown was observed over a wide range of TNF levels, suggesting that the necroptosis biphasic, emergent, and coexistent dynamics were not affected by the intensity of TNF [32]. In line with the experimental observations, our simulations also indicate that when RIP1 is maintained at a normal high level, caspase-8 remains inactive across various TNF intensities (Fig. S2a). Whereas the necroptosis biphasic, emergent, and coexistent dynamics persistently manifest under different TNF intensities (Fig. S2b). Additionally, beyond L929 cells, the phenomenon of necroptosis biphasic and emergent dynamics induced by RIP1 has been observed in other cell types, including mouse embryonic fibroblasts (MEFs) and RAW macrophages [32]. This observation suggests that these dynamics are crucial and universal in cells undergoing necroptosis, influencing the modulation of cell fates.

As RIP3 and C8 can be simultaneously activated (Fig. 1c), coexistence death mode of apoptosis and necroptosis in cells could be triggered by proper RIP1 level. Experimental analysis of cell morphology suggests that only necroptosis occurs in wild-type (WT) cells (100% RIP1), while apoptosis can solely be observed in RIP1 deletion cells (0% RIP1) (Fig. 1h, upper panel). As expected, both necroptosis and apoptosis can be observed in RIP1-impaired cells. The RIP1-induced cell death mode can be well described by potential landscape theory, which provides a more physical description of the stochastic dynamic and global stability of the biological system [33]. Consistent with experimental observations, the middle panel from left to right in Fig. 1h are the landscape topography of RIP1 deletion (single apoptosis state), RIP1 impairment (coexistent state of apoptosis and necroptosis), and WT (single necroptosis state) systems that are mapped in the C8-RIP3 phase space. The kinetic pathway (KP) of the system evolving from random initial state (RIS) in the bottom panel of Fig. 1h visually presents how the decision of death mode is made under different RIP1 levels. With low or high RIP1 level, cells eventually have a unique death mode of apoptosis or necroptosis, while cells have two mode choices with proper RIP1 level, inducing the coexistent dynamics of apoptosis and necroptosis. In addition to the experimental data presented in this article, our previous research and studies by others have revealed the BEC dynamics of RIP1 from various experimental perspectives, including propidium iodide (PI) staining, immunofluorescence microscopy, and mass spectrometry (MS) analysis [11,31,34]. Using these methods, Xiong and his colleagues identified VDAC1 and methamphetamine as crucial regulators in the process of necroptosis [30,35]. Thus, above comparisons confirm that our circuit model has the potential for giving mechanistic insights into the pRIP3/necroptosis BEC dynamics within the TNF-induced death signaling.

### 3.2 RIP1-RIP3-C8 incoherent feedforward loop determines necroptosis BEC dynamics

**Fig. 2:**
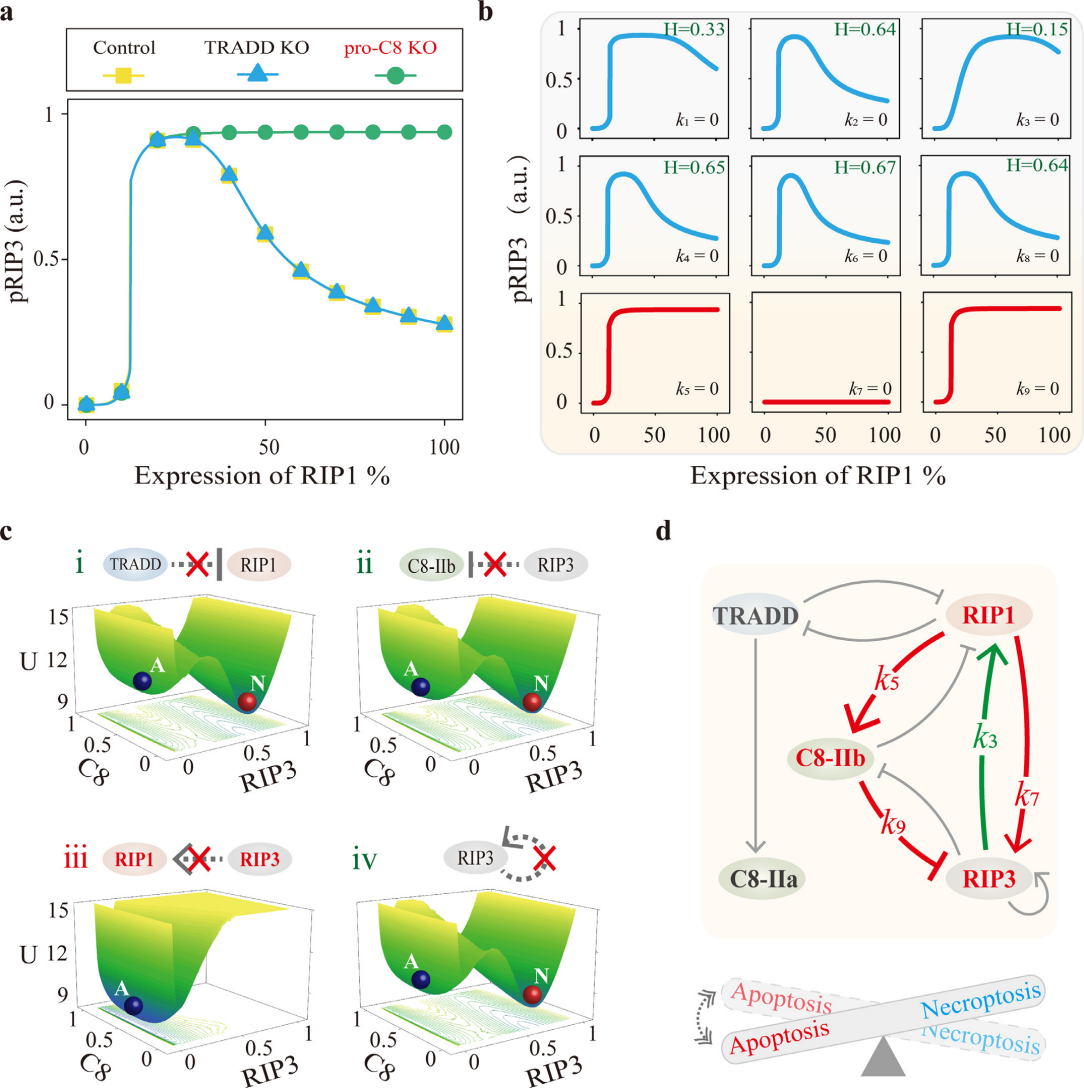
**Identification of the core structure for pRIP3/necroptosis BEC dynamics.** (a) Comparison of pRIP3 BE dynamics under control, TRADD KO, and C8 KO conditions. (b) The dynamics of pRIP3 when any one of the nine interaction terms in circuit model is removed, respectively. (c) Potential landscapes of the system when the four interaction terms are severally removed. (d) Summary of the constituents and terms that can achieve BE dynamics (red lines) with coexistent death mode (green line).

To dissect the essential topology for the BEC dynamics of pRIP3 induced by RIP1, roles of TRADD and C8 are first explored. Biphasic and emergent (BE) dynamics of pRIP3 are not affected by TRADD deletion (Fig. 2a, blue line), but disappear in the absence of C8 (Fig. 2a, green line), implying that C8 is the essential node for BE dynamics. Deletion of C8 barely affects the emergence of pRIP3, and pRIP3 level keeps constant with further increase of RIP1. Thus, the dynamics of pRIP3 consists of two processes: when RIP1 increases from 0 to a low critical level (∼10% RIP1), pRIP3 increases abruptly, inducing the emergent dynamics of necroptosis. With the increase of RIP1, inhibition of C8 on pRIP3 takes effect, causing pRIP3 to gently decrease.

Then, the nine interaction terms among RIP1, RIP3, and C8 in the irreducible circuit model are respectively removed to determine the essential terms (Fig. 2b). BE dynamics disappear when the terms including *k*_5_ (C8 activated by RIP1), *k*_7_ (RIP3 activated by RIP1), and *k*_9_ (RIP3 inhibited by C8) are respectively set to 0, while BE dynamics are still observed when the other six terms are fixed to 0. We introduced the coefficient H, which is defined as *H* = (*pRIP3_Peak_*-*pRIP3_RIP1_100%_*)/*pRIP3_tot_* to quantify the scale of biphasic dynamics. *pRIP3_Peak_* is the maximum level of pRIP3, and pRIP3_RIP1_100%_ is the level of pRIP3 when the expression level of RIP1 is 100% (wild-type). Therefore, analysis in Fig. 2b indicates that the essential topology for necroptosis BE dynamics of the TNF-induced death circuit is constituted by the incoherent feedforward loop structure (*k*_5_, *k*_7_, and *k*_9_) that are embedded in the three nodes (RIP1, RIP3, and C8).

Besides the identified RIP1-RIP3-C8 topological structure for BE dynamics, the interaction term required for achieving the coexistence mode of necroptosis and apoptosis is further explored based on potential landscape analysis. We respectively removed the terms besides the identified essential topology for BE dynamics to determine whether apoptosis and necroptosis states could coexist with RIP1 variation. As shown in Fig. 2c, only when the term of RIP1 activated by pRIP3 (*k*_3_) is removed (Fig. 2c-iii), the system presents solely one potential well with high C8 and low pRIP3 level. However, with the blockade of other terms, the system still exhibits two coexisting wells. Taken together, as the diagram shown in Fig. 2d, RIP1-RIP3-C8 incoherent feedforward loop embedded with the positive feedback of RIP3 to RIP1 is the core topological structure for achieving RIP1-induced necroptosis BEC dynamics.

### 3.3 Bell-shaped regulation of necroptosis biphasic dynamics by RIP3 and the feedforward terms

**Fig. 3:**
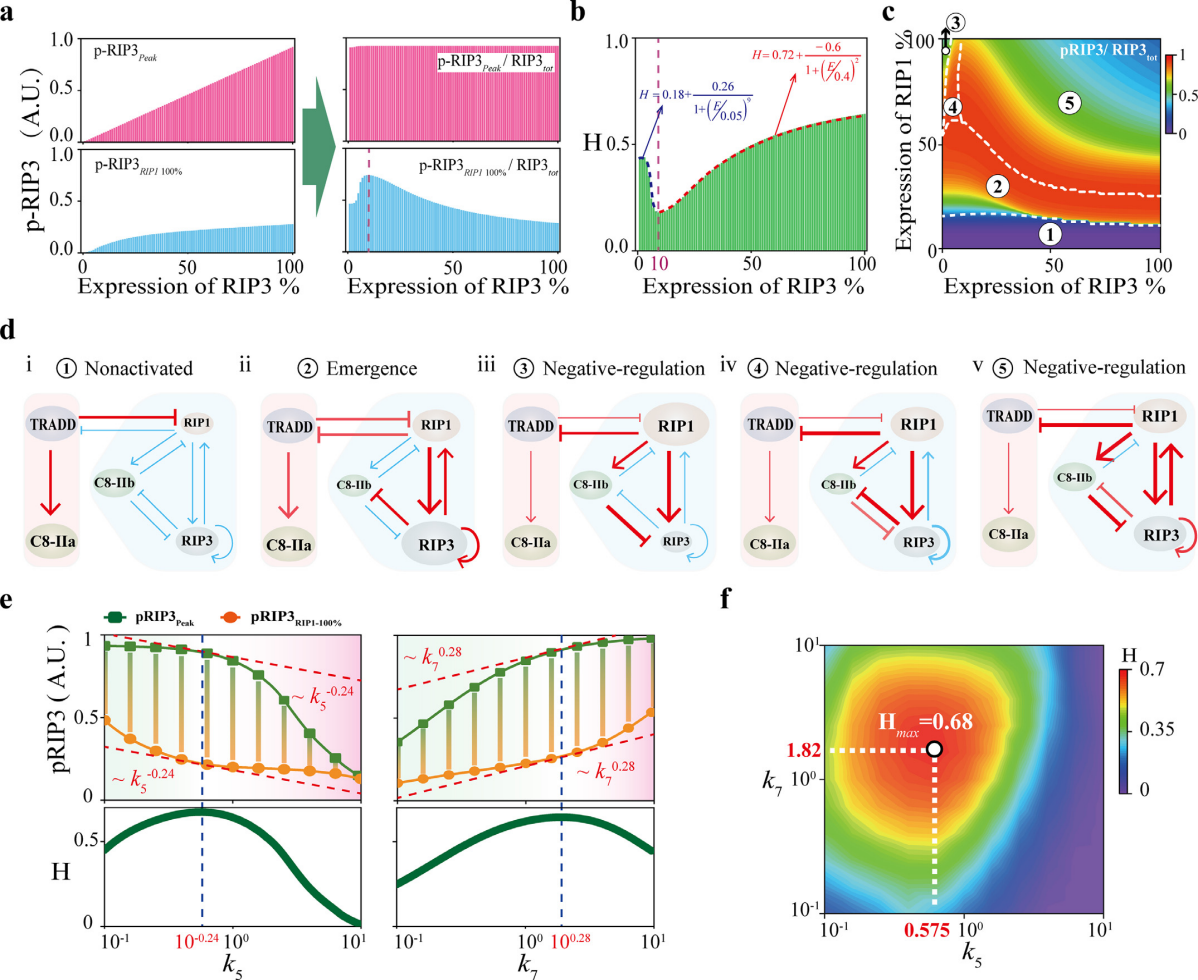
**Scale-free emergence of pRIP3 and bell-shaped regulation of necroptosis biphasic dynamics.** (a) The variation of absolute and relative levels of *pRIP3_Peak_* and pRIP3_RIP1_100%_ with RIP3 expression level increases. (b) The quantified scale H of pRIP3 biphasic dynamics. (c) The relative level of pRIP3 in the RIP3-RIP1 phase plane, giving the plane divided into 5 regions. (d) Topological analysis of the regulatory mechanisms of the 5 regions in (c). (e) Analysis of the *k*_5_ and *k*_7_ bell-shaped regulation on pRIP3 biphasic dynamics. (f) Phase diagram of H in *k*_5_-*k*_7_ parameter spaces.

Having identified the essential topological structure, we next explored the control mechanism of how necroptosis biphasic dynamics is generated and regulated by the protein nodes (RIP3, TRADD, and C8) and interaction terms. Both the absolute and relative levels of *pRIP3_Peak_* and pRIP3_RIP1_100%_ to RIP3 expression level variation are shown in Fig. 3a. The absolute level of *pRIP3_Peak_* is linearly positively correlated with RIP3 (upper left panel of Fig. 3a), whereas the relative level of *pRIP3_Peak_* (*pRIP3_Peak_/RIP3_tot_*) remains constant, presenting a scale-free feature (upper right panel of Fig. 3a). For pRIP3_RIP1_100%_, the absolute level is also positively related to RIP3, but the relative level exhibits a biphasic behavior (down panel of Fig. 3a). The scale-free feature of *pRIP3_Peak_* and biphasic behavior of pRIP3_RIP1_100%_ result in the biphasic dynamics of RIP3 presents inverted bell-shaped responses to RIP3 expression level, as the quantified scale of biphasic dynamics H shown in Fig. 3b. Four-Parameter Logistic Function is considered for a piecewise fit of H. When RIP3 is lower than ∼10%, H decreases monotonically from the maximum value of 0.44 to 0.18, and the decline rate is the largest at ∼5%. Conversely, H is positively correlated with RIP3 when RIP3 is higher than ∼10%. The fitted function suggests that the maximum value of H cannot exceed 0.72 with a maximum increase rate at ∼40%.

To systematically reveal the bell-shaped regulation mechanism of RIP3 on the scale of H, the variation of relative pRIP3 (*pRIP3*/*RIP3_tot_*) is investigated in RIP3-RIP1 phase plane (Fig. 3c). The plane can be divided into 5 regions with specific regulatory mechanisms. Region 1 indicates that the inhibition of TRADD on RIP1 is dominant and RIP3 remains inactive with low expression level of RIP1 (Fig. 3d-i). When RIP1 increases to a critical level, RIP1 activated RIP3 and the self-activation of RIP3 induce the emergence of pRIP3 (Fig. 3d-ii), corresponding to region 2. Regions 3, 4, and 5 show that high level of RIP1 negatively regulates pRIP3. In region 3 with the low expression level of RIP3, pRIP3 is greatly restrained by C8 (Fig. 3d-iii), exhibiting a large value of H. As the expression level of RIP3 increases in region 4, inhibition of pRIP3 on C8 gradually becomes dominant (Fig. 3d-iv). pRIP3 level increases and H decreases. Further increase of RIP3 in region 5 enhances the positive feedback of pRIP3 on RIP1, which indirectly promotes the inhibition of C8 on pRIP3 (Fig. 3d-v), resulting in a resurgence of the large scale of biphasic dynamics (large value of H). We confirmed the inferences though respectively reducing the strength of the corresponding interaction terms, which characterize the mutual inhibition between C8 and pRIP3, and the positive feedback of pRIP3 on RIP1 (Fig. S3a). Specifically, when the strength of C8 inhibition on RIP3 (*k*_6_) decreases, region 3 disappears and the relative level of pRIP3 in region 5 increases. Conversely, weakening the inhibition strength of pRIP3 on C8 (*k*_9_) results in the disappearance of region 4 and the expansion of region 3. Attenuating the positive feedback strength of pRIP3 on RIP1 (*k*_3_) increases the relative level of pRIP3 in region 5. The scale of biphasic dynamics H is barely influenced by TRADD (Fig. S3b), but is gradually enhanced with the increase of C8 (Fig. S3c). For C8, the relative *pRIP3_Peak_* also presents a scale-free feature, while the relative pRIP3_RIP1_100%_ is linearly decreased with the increase of C8.

We next investigated the role of interaction terms in mediating the scale of biphasic dynamics H. Among the nine terms, only *k*_5_ (C8 activated by RIP1) and *k*_7_ (RIP3 activated by RIP1) that involves in the RIP1-RIP3-C8 incoherent feedforward loop, can both mediate the levels of *pRIP3_Peak_* and pRIP3_RIP1_100%_, achieving bell-shaped regulation on H (Figs. 3e and S4). With the increase of *k*_5_, *pRIP3_Peak_* decreases first slow and then fast, while pRIP3_RIP1_100%_ decreases first fast and then slow, presenting distinct responses (Fig. 3e). In contrast, increase of *k*_7_ makes *pRIP3_Peak_* to increase first fast and then slow, but pRIP3_RIP1_100%_ to increase first slow and then fast. The scale H is the largest when the change rate of *pRIP3_Peak_* and pRIP3_RIP1_100%_ are equal with the corresponding strengths *k*_5_ = 10^−0.24^ and *k*_7_ = 10°^.28^. *pRIP3_Peak_* can hardly be regulated by the rest seven terms, while pRIP3_RIP1_100%_ is positively regulated by *k*_6_ and negatively regulated by *k*_1_, *k*_3_, and *k*_9_ (Fig. S4a). Further two-parameters phase plane analysis indicates that the maximum value of H is 0.68 when *k*_5_ = 0.575 and *k*_7_ = 1.82 (Fig. 3f). We severally profiled *pRIP3_Peak_* and pRIP3_RIP1_100%_ in the *k*_5_-*k*_7_ phase plane (Fig. S5a), and three different regions and two processes are identified in the plane to reveal the mechanism of bell-shaped regulation (Fig. S5b). Although the term of *k*_9_ (inhibition of C8 on pRIP3) that involves in the RIP1-RIP3-C8 incoherent feedforward loop can not drive bell-shaped regulation on H, *k*_9_ significantly amplifies the bell-shaped regulation of *k*_5_ (C8 activated by RIP1) or *k*_7_ (RIP3 activated by RIP1) on H (Fig. S5c).

### 3.4 Shannon entropy quantifies uncertainty of the coexistence death modes

**Fig. 4:**
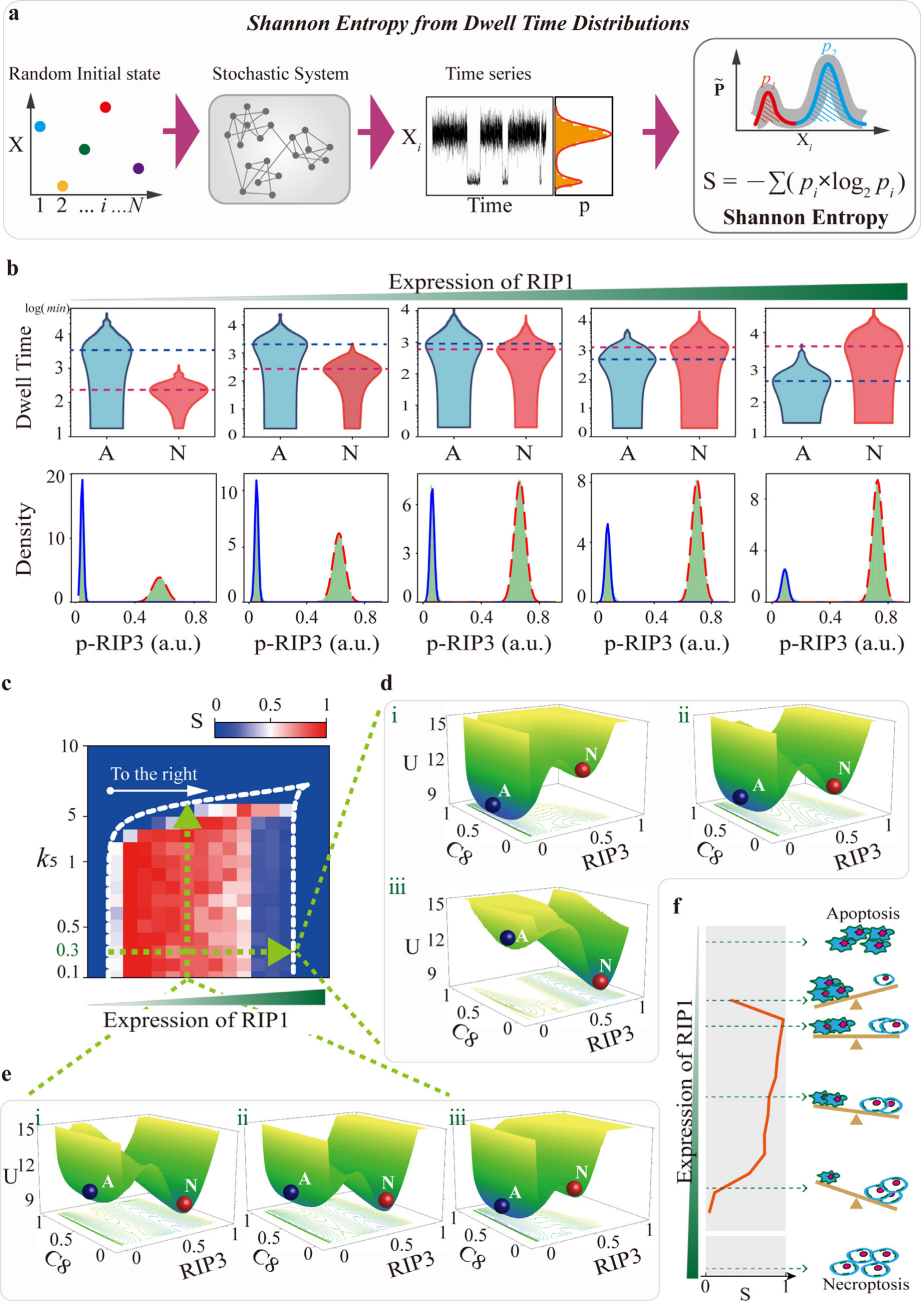
**Shannon entropy quantifies the uncertainty of cell fate decisions.** (a) Illustration of the calculation procedure of Shannon entropy with dwell time distribution. (b) Statistics and distribution of dwell time under five representative RIP1 expression levels at the free degrees of pRIP3. (c) The quantified Shannon entropy of k_5_ in regulating RIP1-dependent coexistent dynamics. (d) and (e) The potential landscape topography of coexistent death modes with *k*_5_ = 0.3, and RIP1 level at 10%, 11%, and 12.5% respectively (d), and the level of RIP1 at 11% with *k*_5_ = 0.1, 0.5, and 0.7 respectively (e). (f) Shannon entropy characterizes the uncertainty of cell fate, and a diagram of “seesaw” that reflects the death modes decision under different RIP1 levels.

Previous studies attempted to infer the information for thermodynamic quantities by observing dwell time distribution of transitions among multiple steady states [[36], [37], [38]]. The cell death signaling presents coexistent dynamics with proper RIP1 level, as the two basins (apoptosis and necroptosis) shown in the death landscape topography (Fig. 1g). To measure the uncertainty of cell fate decision, we introduced Shannon entropy to estimate entropy production, which is defined as *S* = -Σ*p_i_log*_2_(*p_i_*). *p_i_* is the probability of the *i* th death mode, and *i* represents the state of apoptosis or necroptosis. S characterizes the degree of disorder of the cell death system. The larger the value of S is, the more disorder the system is. Fig. 4a illustrates the analysis procedure for Shannon entropy by calculating dwell time distribution. Stochastic cell death system is obtained by adding Gaussian white noise to the deterministic system with random initial states. Temporal dynamics of the system are precisely recorded at the free degrees of pRIP3 to obtain the dwell time distribution of the system in different states. With the increase of RIP1, the dwell time in apoptosis state is gradually decreased, but is increased in necroptosis state (Fig. 4b, upper panel), and the corresponding dwell time distributions are shown as well (Fig. 4b, down panel).

Shannon entropy of *k*_5_ (term of C8 activated by RIP1 that exhibits bell-shaped regulation on necroptosis biphasic dynamics) in regulating RIP1-dependent coexistent dynamics is measured (Fig. 4c). The region surrounded by the white dotted line is the coexistence transition region. The color code in the transition region indicates the degree of transition between apoptosis and necroptosis of the system. The system presents a highly disordered state when RIP1 is near the level to induce pRIP3/necroptosis emergent dynamics. Shannon entropy (S) gradually decreases, and necroptosis becomes dominated with further increase of RIP1. Thus, the cell fate switches from ordered apoptosis to highly disordered and finally to ordered necroptosis with the increase of RIP1. A more intuitive potential landscape topography transition is shown in Fig. 4d when *k*_5_ is fixed at the standard value of 0.3. With the increase of RIP1, the depth of the apoptosis basin is gradually decreased, while the necroptosis basin is increased, suggesting that RIP1 biases the cell fate towards necroptosis. The transitions of Shannon entropy and potential landscape topography with different *k*_5_ are also investigated with RIP1 is fixed at 11% (Fig. 4e). In contrast to RIP1, increase of *k*_5_ results in the uncertainty of cell fate switches from highly disordered to ordered apoptosis, giving the depth of apoptosis basin increased and necroptosis basin decreased. The entropy result also reveals that the increase of *k*_5_ causes a high-level demand for RIP1 to trigger pRIP3 emergent dynamics (white arrow in Fig. 4c). The larger the value of *k*_5_ is, the higher RIP1 level is required for inducing necroptosis emergent dynamics.

Therefore, acting as the driving force, RIP1 makes the system dynamics like a “seesaw” (Fig. 4f). The system exclusively executes apoptosis at low RIP1 level. Increase of RIP1 significantly elevates pRIP3 level and entropy production. The death system is highly disordered and will selectively undergo apoptosis or necroptosis, depending on the initial conditions. High level of RIP1 reduces entropy production and drives the system to ordered necroptosis. RIP1-dependent coexistent dynamics regulated by the other three reactions within the identified essential topological structure (Fig. 2d) are shown in Fig. S6, indicating that the terms of *k*_3_ (RIP1 activated by pRIP3) and *k*_7_ (RIP3 activated by RIP1) can efficiently switch death modes, while the system remains highly disordered with the variation of *k*_9_ (inhibition of C8 on pRIP3).

### 3.5 Random circuit analysis identifies two core topologies for necroptosis BEC dynamics induction

**Fig. 5:**
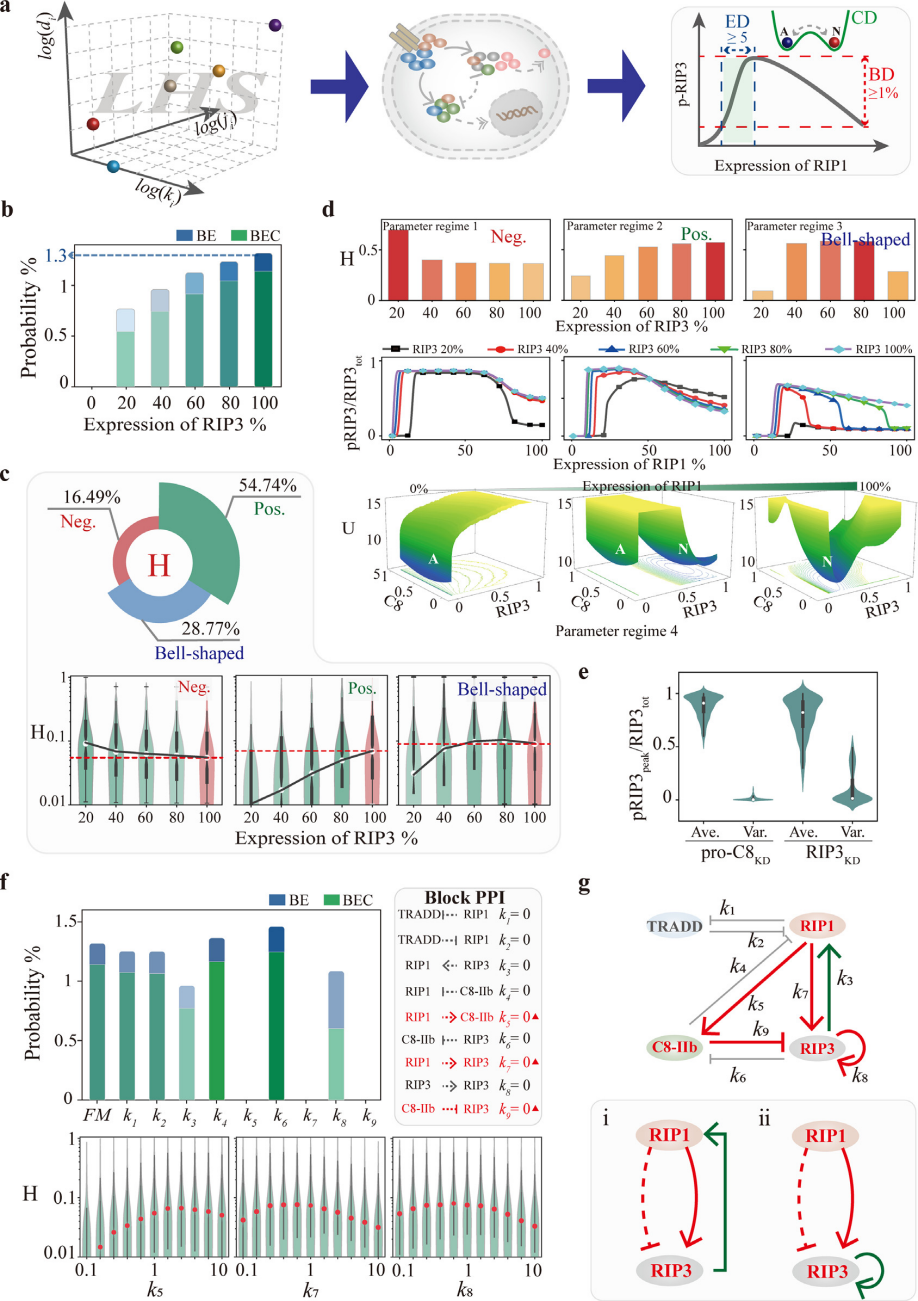
**Identifying the core structure for necroptosis BEC dynamics with random circuit analysis.** (a) Schematic of a computational workflow. Left panel: Latin hypercube sampling to obtain random parameter regimes. Right panel: Threshold settings for screened RIP1-dependent pRIP3 dynamics, including biphasic, emergent, and coexistent dynamics (BD, ED, and CD). (b) Random circuit analysis is used to search 50,000 systems under five representative RIP3 expression levels and count the probabilities that pRIP3 dynamic behaviors satisfy the threshold condition of BE and BEC dynamics. The blue and green histograms represent the probability of BE and BEC dynamics, respectively. (c) Statistics of the regulatory behavior of RIP3 on H in the screened systems with BEC dynamics. (d) Four representative systems of three different regulations of RIP3 on H (parameter regimes 1–3) and RIP1-dependent death modes switching behavior (parameter regimes 4). e) The average and variance statistics of pRIP3peak relative level of all the screened samples with different expression levels of RIP3 and C8. (f) The probabilities of the system achieving BE (blue histograms) and BEC dynamics (green histograms) when any one term is removed. The terms of *k*_5_, k_7_, and k_9_ (marked by red triangles) are individually removed and the system cannot achieve biphasic dynamics. Down panels: Statistics of terms strength on H in the screened systems. (g) The components and reactions in the death circuit to achieve BEC dynamics. Red lines indicate the necessary interaction of the incoherent feedforward loop. Green lines indicate the positive feedback to RIP3.

To avoid the specificity inherent in deterministic models with specific parameter regimes, random circuit analysis is further performed to identify the core topology for necroptosis BEC dynamics. Latin hypercube sampling is used to obtain uniform random parameter regimes within reasonable biological interval [11,13,19], and all parameter regimes that can achieve BE, and BEC dynamics are screened (Fig. 5a). We employed a random sampling range for parameter regimes within a biologically plausible spectrum. Distinct parameter regimes correspond to different cells, potentially originating from entirely distinct cell lineages. We assumed that the biphasic dynamics (BD) of pRIP3 satisfies *pRIP3_Peak_* is higher than pRIP3_RIP1_100%_ by more than 1%, i.e., BD is defined when BD = (*pRIP3_Peak_* - *pRIP3_RIP1_100%_*) / *pRIP3_Peak_* ≥ 1%. Since pRIP3 presents an abrupt and large increase at low level of RIP1 (Fig. 2a), emergent dynamics (ED) is also considered through satisfying pRIP3 is increased by more than 50% when RIP1 continuously increases by 10% (ED = Δ*pRIP3*/Δ*RIP1* ≥ 5).

The probabilities under five representative RIP3 expression levels are firstly calculated, and 50,000 groups of random samples are taken for each RIP3 level. The probabilities of achieving BE dynamics (blue histograms) and BEC dynamics (green histograms) are presented in Fig. 5b. As the result indicated, the vast majority (∼86.6%) of samples that achieve BE also have coexistent dynamics, revealing that the necroptosis BE dynamics is highly related to coexistence of death mode. The probabilities of BE and BEC dynamics decrease monotonically with RIP3 decreases. The probability of only considering biphasic dynamics is decreased by 17% when RIP3 decreases to 20% (Fig. S7a). Whereas, the probability of BEC dynamics is decreased by 41.6% (Fig. 5b). Thus, compared to biphasic, RIP3 seems to be more critical for the achievement of emergent and coexistent dynamics.

To determine the regulation of RIP3 on the scale of biphasic dynamics H, 570 samples that achieve BEC dynamics for 100% RIP3 are selected. The regulation of H by RIP3 has three types and their corresponding proportions are calculated: negative regulation (H is decreased with RIP3 increases) with 16.49%, positive regulation (H is increased with RIP3 increases) with 54.74%, and bell-shaped regulation (nonlinear change in H with RIP3 increase) with 28.77% (Fig. 5c). Three specific systems are further selected to intuitively show how RIP3 negatively, positively, or nonlinearly regulates H (Fig. 5d, upper and middle panels). Another specific system is also selected to present the death mode transitions from apoptosis to the coexistence of apoptosis and necroptosis, and finally to the exclusive necroptosis state with the increase of RIP1 (Fig. 5d, down panel).

Similar regulation of C8 on H is shown in Fig. S7b, where the proportion of systems that achieving bell-shaped regulation of H by C8 is less than ∼10% compared to the regulation of RIP3 on H (Fig. 5c). Thus, variation of RIP3 seems to be more easily than C8 to achieve bell-shaped regulation on H, supporting the results in the deterministic system that H does not present bell-shaped response to C8 variation (Fig. S3c). The average and variation of *pRIP3_peak_* with the decrease of RIP3 or C8 in the 570 systems are calculated as well (Fig. 5e). The average of relative *pRIP3_peak_* is concentrated at a high level and the variation is quite small, revealing that the scale-free feature of necroptosis emergence (Fig. 3a) is an intrinsic topological property of the death circuit.

The essential module for achieving BE and BEC dynamics are discussed with random circuit analysis as well. The interaction terms are completely blocked one by one in the 50,000 random samples, and the statistical probabilities are shown in Fig. 5f. Only when any one of the terms (*k*_5_, *k*_7_, or *k*_9_) is removed, the probability tends to be 0, suggesting that the RIP1-RIP3-C8 incoherent feedforward loop is the necessary module to generate BE and BEC dynamics (Fig. 5f and S7c). Positive feedback loop is proven to be necessary for coexistent dynamics [39]. Unlike the deterministic system (Fig. 2c), blocking the positive feedback of RIP3 to RIP1 (*k*_3_) cannot arrest coexistent dynamics (Fig. 5f). However, the probability of BEC is significantly decreased compared to BE with the blockage of RIP3 self-activation (*k*_3_). The contributions of all the four positive feedback loops within the death circuit are studied (Fig. S7d), revealing that only the positive feedback loop formed by *k*_3_ or *k*_8_ is efficient for achieving the high occurrence probability of coexistent dynamics.

The regulation strategies of H by all the circuit interaction terms are separately calculated (Fig.s 5f and S7e). Consistent with deterministic system analysis (Fig. 3e), the bell-shaped regulation of H by the two feedforward terms of *k*_5_ and *k*_7_ are also statistically confirmed (Fig. 5f). Moreover, RIP3 self-activation term *k*_8_ can also present bell-shaped regulation on H, which is not observed in the deterministic system (Fig. S4b). Thus, besides the positive feedback of RIP3 to RIP1 (Figs. 5g-i and 2d), RIP3 self-activation forms another positive feedback for efficiently achieving coexistent dynamics and the bell-shaped regulation on necroptosis biphasic dynamics (Fig. 5g-ii). The RIP1-RIP3-C8 incoherent feedforward loop embedded with these two positive feedback loops forms the fundamental hypermotif for robustly achieving BEC dynamics in the death circuit (Fig. 5g).

### 3.6 Topological exhaustivity defines three minimal circuits to achieve natural BEC dynamics

To dissect the hidden design principles in biological systems, we tried to find the minimal circuit that performs biphasic dynamics with emergence. Topological exhaustive method has been widely used to explore the design of functional achievement in biological networks [13,14,40,41]. The workflow for circuit topology to function mapping of BE dynamics is shown in Fig. S8a, which presents the circuit model described by coupling matrices, parameter regimes, and ordinary differential equations. We first examined whether the output signal node in the two-node circuit could achieve BE dynamics by the variation of the receiving node of input. All the 27 different structures of two-node circuit are respectively assigned 100,000 sets of random parameter regimes, but none of these circuits can achieve BE dynamics (Fig. S8b).

**Fig. 6:**
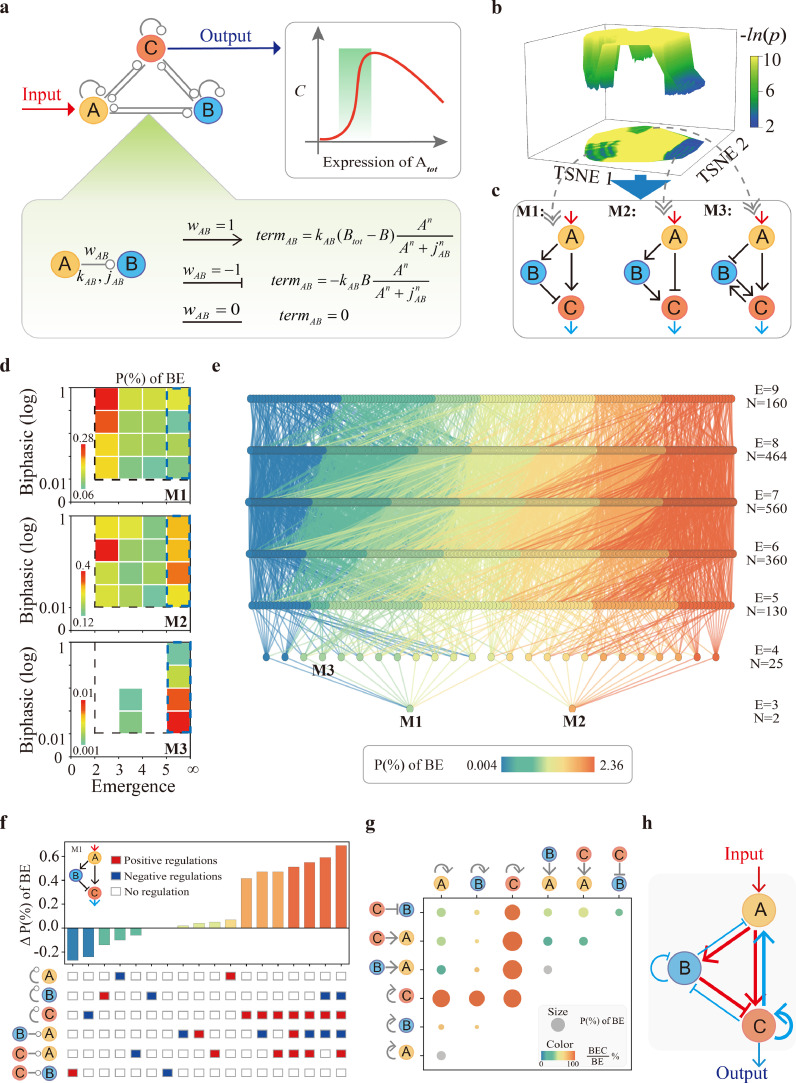
**Topological exhaustive method reveals a complete atlas of achieving BEC dynamics in three-node circuit.** (a) Illustration of the structure screening procedure from mapping topology to function of asymmetric directed three-node circuit. A, B and C are the input node, regulatory node, and output node, respectively. There are three kinds of edges between any two nodes, *w* = 1 means promotion, *w* = −1 means inhibition, and *w* = 0 means no interaction. (b) Topological landscape of 4698 coupling matrices in a 2D topological space. The well depth represents the probability of a sub-cluster achieving BE dynamics. (c) The minimal circuit of the sub-clusters that corresponds to the three wells in the topological landscape. (d) Probability distributions of the three minimum circuits are mapped into the biphasic dynamics and emergence 2D scale spaces. (e) A global atlas of 1,701 circuits that enable topological evolution of three-node circuits for achieving BE dynamics. (f) Probability statistics of BE dynamics that can be achieved by adding edges based on circuit M1. (g) The proportions of systems that with BEC dynamics when the six positive feedback edges are added to M1 individually (diagonal node) or in combination (non-diagonal node). (h) The determined optimal circuit for robustly achieving BEC dynamics.

We next searched BE dynamics in the three-node circuit, which includes an input node (node-A), an output node (node-C), and a regulatory node (node-B) (Fig. 6a). There are three types of term (promotion, inhibition, and no interaction) between any two nodes. To map out the entire design space of three-node circuits capable of BE dynamics, all the 4,698 circuits are exhausted and analyzed with 50,000 sets of random parameter regimes assigned to each circuit. A total of 234.9 million dynamical systems (4,698 × 50,000 parameter regimes) are analyzed and finally 1,701 circuits that can achieve BE dynamics are screened out. To quantify the volume of the parameter space in which circuit supports BE dynamics, the 4,698 coupling matrices are firstly reduced to two-dimensional space using *t-SNE* method [42], and then the probabilities are converted into topological potentials through -ln(*p*) analogy to the Boltzmann relation (Fig. 6b). As a result, the three potential wells in the topological landscape correspond to three sub-clusters respectively. The three minimal circuits (M1, M2, and M3) in each sub-cluster are shown in Fig. 6c, indicating that the circuits have the common feature of containing incoherent feedforward loop. Similar results are obtained with clustering analysis using the pair-wise distance between circuits, which also divides the 1,701 circuits into three sub-clusters and finally refers to the same three minimal circuits (Fig. S9a).

Then, we severally segmented the obtained probabilities of the three sub-clusters into the two-dimensional space of biphasic and emergent dynamics (Fig. 6d). Probability distributions of the three sub-clusters are quite different in the scale space. The sub-cluster of M1 circuit has a high probability occurrence with a large scale of biphasic dynamics but with a low scale of emergent dynamics (red square). Additionally, the sub-cluster of M2 circuit prefers to achieve a middle scale of biphasic dynamics but with a small or high scale of emergent dynamics (red and orange squares). M3 circuit sub-cluster is concentrated on attaining a small scale of biphasic dynamics, but with a high scale of emergent dynamics (red square). Therefore, despite containing the incoherent feedforward loop, the three minimal circuits exhibit divergence scales for achieving BE dynamics, providing potential diversity control strategies in regulating various biological functions.

Starting from the three minimal circuits, the 1,701 circuits that can achieve BE dynamics are generated by adding edges one by one. As a result, a comprehensive atlas describing the topological evolution of three-node circuits and their corresponding probabilities for achieving BE dynamics are entirely presented in Fig. 6e. The topological complexity (the number of interaction terms/edges E) of the atlas increases from bottom to top, and the probability of a circuit of the same complexity to achieve BE dynamics decreases from left to right. The connectivity in the global atlas of topological evolution could supervise any one interaction to enhance or resist the circuit fulfillment function.

If an added edge improves the probability for achieving BE dynamics of the minimal circuit, such an edge is functionally significant [19]. The statistical result of stochastically adding edges to improve the probability of achieving BE dynamics based on the minimum motif M1 is shown in Fig. 6f. The result indicates that adding the self-activation of node C significantly increases the probability for inducing BE dynamics. The probability is decreased by adding inhibition, but is increased through considering the promotion of node C on node A. Based on the structure of adding node C self-activation and the promotion of node C on node A, the probability reaches the highest while further considering the inhibition of node B on node A and self-inhibition of node B in M1. Positive feedback loop is proved to play decisive roles for realizing multi-stable states in biological systems [39]. To discuss the structure of M1 for robustly achieving coexistent dynamics, six terms which could form positive feedback loops in motif M1, are individually (diagonal node) or integratively (non-diagonal node) added (Fig. 6g). The proportion of the system with BEC dynamics based on the achieved BE dynamics system is calculated, showing that the contribution of node C self-activation is significant, and the proportion of system with coexistent dynamics is the highest when the promotion of node C on node-A is added.

Taken together, for robustly achieving BEC dynamics, the structure shown in Fig. 6h should be the optimal circuit topology for M1, where red lines represent the essential edges while blue lines are the regulatory edges. Actually, the experimentally determined RIP1-RIP3-C8 circuit (Fig. 1b) is highly consistent with the screened optimal circuit, revealing the precise design strategy of the biological system in controlling cell death. The optimal structure for circuits M2 and M3 are also discussed and the corresponding results are shown in Fig. S9b, S9c.

## 4 Discussion

To explore the topology and regulatory mechanism for necroptosis biphasic, emergent, and coexistent dynamics, we proposed a circuit cell death model of the TNF signaling based on previous studies [11] and our experimental data (Fig. 1). We determined that the RIP1-RIP3-C8 incoherent feedforward loop is necessary for achieving biphasic dynamics with emergence, while the positive feedback loop of RIP3 on RIP1 is required for death mode coexistence (Fig. 2d). Instead of exploring the mechanisms with specific models, random parameter analysis of the TNF circuit is also performed, identifying that the incoherent feedforward loop embedded with RIP3 self-activation is another effective structure for achieving BEC dynamics (Fig. 5g). We attempted to explore whether there exist general circuit design principles for natural systems to execute BEC dynamics by using the topological landscape (Fig. 6b), bottom-up, and topological evolution (Fig. 6e) strategies. Both two- and three-node circuits are systematically analyzed and only three minimal three-node circuits are identified finally, confirming that the incoherent feedforward loop is the essential module for BEC dynamics.

**Fig. 7:**
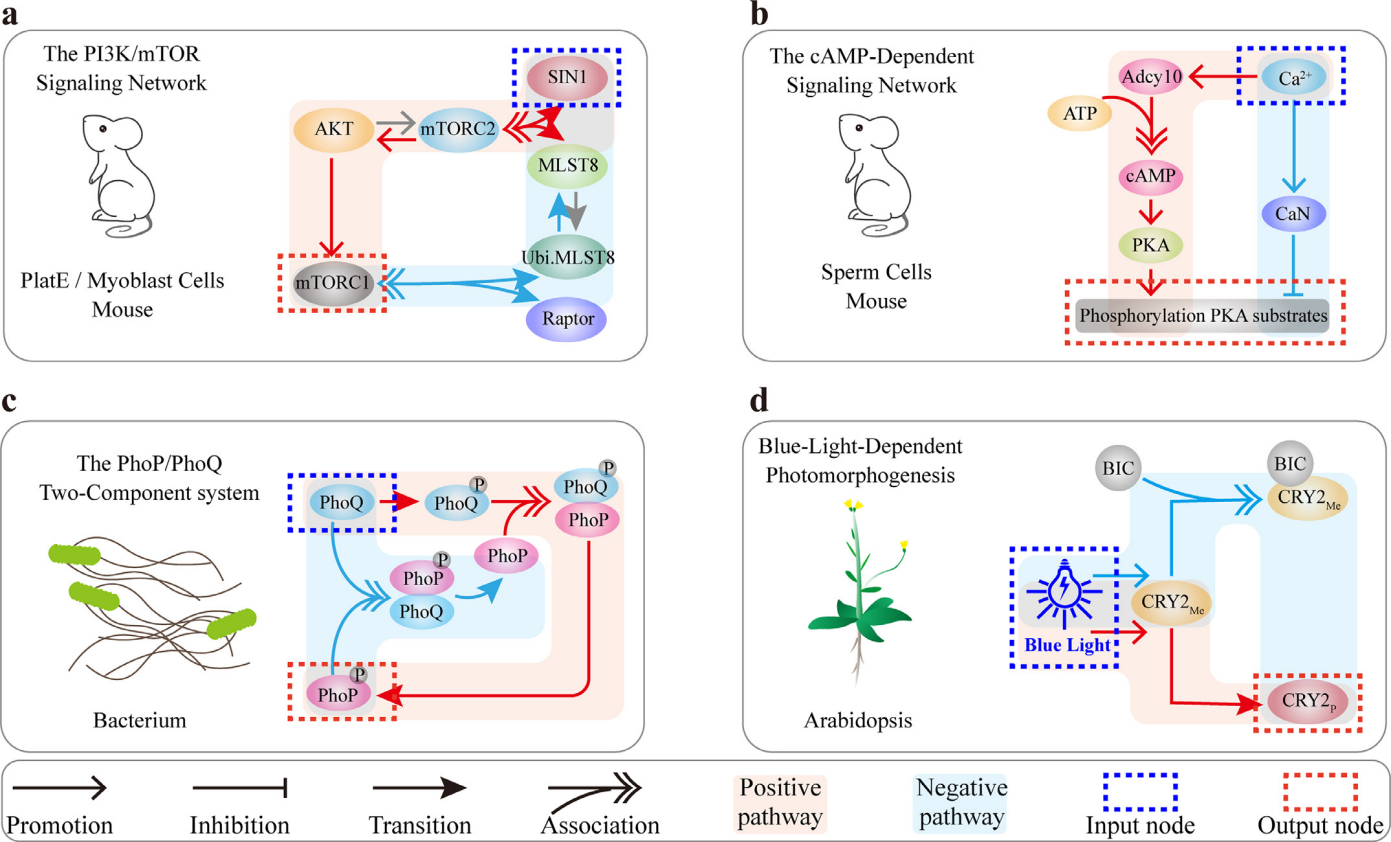
**Examples of biphasic dynamics induced by incoherent feedforward loop in various cell types.** Blue backgrounds represent the negative regulatory pathway, and red backgrounds represent the positive regulatory pathway. (a) Biphasic dependence of mTORC1 activity on SIN1 in mTOR signaling. (b) Biphasic function of calcium ions in cAMP-dependent signaling. (c) The dual role of PhoQ in regulating PhoP phosphorylation in bacterial two-component system. (d) Biphasic response of cryptochrome photoreceptor 2 (CRY2) to photomorphogenesis in Arabidopsis.

Biphasic dynamics have been observed to drive essential biological processes in all forms of life, including mammalian cells, plant cells, and even bacterial cells. Previous study reported that SIN1, a key mTORC2 subunit, biphasically regulates mTORC1 activity in Myoblast cells [43]. The dynamics is determined by the incoherent feedforward loop shown in Fig. 7a. Low-dose SIN1 promotes mTORC1 by synthesizing mTORC2, whereas high-dose SIN1 over-depletes MLST8 resulting in mTORC1 decreases. Biphasic dynamics is also observed in cAMP signaling which is triggered by the incoherent feedforward loop [44] (Fig. 7b). Calcium ions positively regulate Adcy10 to promote cAMP synthesis and PKA activation in sperm flagellum, and also inhibit PKA by activating CaN (calcineurin). Besides the mammalian cells, we previously found the biphasic dynamics of PhoP phosphorylation regulated by PhoQ in bacteria [6]. The incoherent feedforward loop exists in the PhoP/PhoQ signaling as well (Fig. 7c). On the one hand, PhoQ promotes PhoP phosphorylation through binding to PhoP. On the other hand, excess unphosphorylated PhoQ also binds to phosphorylated PhoP to dephosphorylate PhoP. Our former study also observed the biphasic dynamics of CRY2 controlled by blue light in Arabidopsis [8], and the incoherent feedback loop is also hidden in the CRY2 signaling (Fig. 7d). With the increase of light intensity, blue light not only promotes the transition of CRY2_Me_ to CRY2_p_, but also promotes the combination of CRY2_Me_ and BIC to form a complex to reduce the level of CRY2_p_. These findings suggest that the incoherent feedforward loop should be a generalizable design principle for robustly executing biphasic dynamics in biological systems.

Despite the complexity and diversity of cell signaling networks, their core module and central topology should be highly conserved. Understanding the general design principles to achieve specific functions in diverse biological systems is significant. Forward searching all two- or three-node circuits are effective to find essential structures for achieving functions such as adaptation, noise-attenuation, robust oscillation, *etc.* [[13], [14], [40], [45]],. The biological systems frequently exhibit muti-functions at the same time. Unlike the previous studies that mainly focused on one or two biological functions, we identified the topological structure that can achieve three general functions, i.e., biphasic, emergent, and coexistent dynamics in this work. Among the identified circuits, auxiliary interaction on M1 motif that increase the probability of functional achievement are consistent with the experimentally observed RIP1-C8-RIP3 structure (Figs. 2d, 6c), suggesting that the biological systems are naturally optimal structures. Of course, topological exhaustivity is also a powerful approach for predicting interaction in biological systems that have not been experimentally observed. In our analysis, the three identified circuits (M1, M2, and M3) seem to exhibit divergence scales for achieving BE dynamics (Fig. 6d). However, the intrinsic differences among these circuits are not captured. Further studies are still needed to systematically compare the general principles of these incoherent feedforward loops in exerting biological functions.

Cell states correspond to the attractors of the dynamical system, while potential landscape captures the dynamical principles of cell state transitions through providing a global characterization and stability measurement [[46], [47], [48]]. Potential landscape allows the targeted exploration of fundamental features and switching strategies of cell fate decision processes, and their application deepens our understanding of biological functions [49]. This is the first landscape discussion of necroptosis signaling to investigate the regulation of death mode switching. We systematically explored how the system structure changes the volume of the valleys, potentially helping to develop therapeutic strategies for death control. However, when employing the landscape theory, it is still difficult to use Fokker-Planck equation to solve the evolution probability of high-dimensional complex system. Although it has been proven effective to coarse-grain a high-dimension system into a low-dimension [50], the curse of dimensionality exists objectively. Thus, deep learning method, truncated moment equations, partial self-consistent mean field approximation, and trajectory density should be developed and further considered in future studies [50,51].

For living systems with nonequilibrium multi-stable states, the essence of state switching is violating the principle of detailed balance that occurs at the cost of increasing entropy [52]. However, the complexity and only partial accessibility of living systems severely limit the inference of crucial thermodynamic quantities, like the entropy production. Previous theoretical explorations provided groundbreaking insights into understanding the central dogma, cells sense through receptors, and so on [53,54]. In this study, Shannon entropy is introduced for the first time to measure the uncertainty of cell death mode transition. Information entropy presents a possible paradigm for understanding the transformation of energy and information in cells to perform fate decisions. However, due to the macroscopic limitation of complex living systems, this work assumes that the state transitions on the observed degrees of freedom are equivalent to the state transitions of all degrees of freedom of the system. We cannot determine whether there are other state transitions based on partial observations. Inferring information and energy associations in living systems remains a significant challenge.

## Declaration of competing interest

The authors declare that they have no conflicts of interest in this work.

## Appendix Supplementary materials

Image, application 1
