# Supplementary material for: Incoherent feedforward loop dominates the robustness and tunability of necroptosis biphasic, emergent, and coexistent dynamics
Source: Fundam Res. 2024 Mar 5;6(2):691–704. doi: 10.1016/j.fmre.2024.02.009 (PMC13069638; doi:10.1016/j.fmre.2024.02.009)
Supplement: Supplementary file 1 [file mmc1.pdf]

Supporting Information

Incoherent feedforward loop dominates the robustness and tunability of necroptosis biphasic, emergent, and coexistent dynamics

Fei Xu *et al.*

☐ Computational modeling

Table 1 The complete equations of deterministic TNF circuit model.

ODEs	Interaction term	Parameters
$\frac{d[acTRADD]}{dt} = F_{TRADD}$ $F_{TRADD} = v_1 - v_2 - v_3$	Activation by *TNF*	k_{ac1}=1.7 j_{ac1}=0.012 n_{ac1}=3
	$v_1 = k_{ac1} * (1 - [acTRADD]) * \frac{[TNF]^{n_{ac1}}}{([TNF]^{n_{ac1}} + j_{ac1}^{n_{ac1}})}$	
	Inhibition by *pRIP1*	k_1=9.5 j_1=0.12 n_1=2
	$v_2 = k_1 * [acTRADD] * \frac{[pRIP1]^{n_1}}{([pRIP1]^{n_1} + j_1^{n_1})}$	
	Degradation	d_1=0.03
	$v_3 = d_1 * [acTRADD]$	
$\frac{d[pRIP1]}{dt} = F_{RIP1}$ $F_{RIP1} = v_4 + v_5 - v_6 - v_7 - v_8$	Activation by *TNF*	k_{ac2}=0.15 j_{ac2}=2.3 n_{ac2}=1
	$v_4 = k_{ac2} * (1 - [pRIP1]) * \frac{[TNF]^{n_{ac2}}}{([TNF]^{n_{ac2}} + j_{ac2}^{n_{ac2}})}$	
	Phosphorylation by pRIP3	k_3=6.7 j_3=1.2 n_3=3
	$v_5 = k_3 * (1 - [pRIP1]) * \frac{[pRIP3]^{n_3}}{([pRIP3]^{n_3} + j_3^{n_3})}$	
	Inhibition by *acTRADD*	k_2=0.17 j_2=1.47 n_2=4
	$v_6 = k_2 * [pRIP1] * \frac{[acTRADD]^{n_2}}{([acTRADD]^{n_2} + j_2^{n_2})}$	
	Cleavage by *C8* in Complex-IIb	k_4=1.4 j_4=0.008 n_4=4
	$v_7 = k_4 * [pRIP1] * \frac{[C8_{IIb}]^{n_4}}{([C8_{IIb}]^{n_4} + j_4^{n_4})}$	
	Degradation	d_2=0.1
	$v_8 = d_2 * [pRIP1]$	
$\frac{d[pRIP3]}{dt} = F_{RIP3}$ $F_{RIP3} = v_9 + v_{10} - v_{11} - v_{12}$	Phosphorylation by *pRIP1*	k_7=2.1 j_7=0.16 n_7=4
	$v_9 = k_7 * (1 - [pRIP3]) * \frac{[pRIP1]^{n_7}}{([pRIP1]^{n_7} + j_7^{n_7})}$	
	Autophosphorylation	k_8=1.0 j_8=11.4 n_8=4
	$v_{10} = k_8 * (1 - [pRIP3]) * \frac{[pRIP3]^{n_8}}{([pRIP3]^{n_8} + j_8^{n_8})}$	
	Cleavage by *C8* in Complex-IIb	k_9=8.5 j_9=0.0015 n_9=4
	$v_{11} = k_9 * [pRIP3] * \frac{[C8_{IIb}]^{n_9}}{([C8_{IIb}]^{n_9} + j_9^{n_9})}$	
	Degradation	d_3=0.14
	$v_{12} = d_3 * [pRIP3]$	
$\frac{d[C8]}{dt} = F_{C8_{IIa}} + F_{C8_{IIb}}$ $F_{C8_{IIa}} = v_{13} - v_{16}$ $F_{C8_{IIb}} = v_{14} - v_{15} - v_{16}$	Activation by *acTRADD* in Complex-IIa	k_{10}=3.6 j_{10}=1.25 n_{10}=4
	$v_{13} = k_{10} * (1 - [C8]) * \frac{[acTRADD]^{n_{10}}}{([acTRADD]^{n_{10}} + j_{10}^{n_{10}})}$	
	Activation by *pRIP1* in Complex-IIb	k_5=0.3

	$v_{14} = k_5 * (1 - [C8]) * \frac{[pRIP1]^{n_5}}{([pRIP1]^{n_5} + j_5^{n_5})}$	j_5=10.4<br>n_5=2
	Inhibition by *pRIP3* in Complex-IIb	k_6=0.2<br>j_6=0.036<br>n_6=4
	$v_{15} = k_6 * [C8_{IIb}] * \frac{[pRIP3]^{n_6}}{([pRIP3]^{n_6} + j_6^{n_6})}$	
	Degradation	d_4=0.35
	$v_{16} = d_4 * [C8]$	

☐ Kinetic parameters estimation

Table 2 Public experimental data sources for parameter estimation [1].

This paper	Data Sources	
Figure 1d	Figure 5A, 5F and 5H	https://doi.org/10.1007/s13238-020-00810-x
Figure 1e	Figure 3F, 5F and S3B	
Figure 1g	Figure 5H	

☐ Abbreviation list

Table 3 List of abbreviations and acronyms

**BEC**	Biphasic, Emergent, and Coexistent
**TNF**	Tumor necrosis factor
**TNFR1**	Tumor necrosis factor receptor 1
**TRADD**	TNFR1-associated death domain protein
**RIP1**	receptor-interacting protein kinase 1
**RIP3**	receptor-interacting protein kinase 3
**C8**	caspase-8
**RSK**	ribosomal S6 kinase
**RHIM**	RIP Homotypic Interaction Motif
**MLKL**	mixed lineage kinase domain-like protein
**ERK**	extracellular regulated protein kinases
**ADP**	adenosine diphosphate
**AMP**	adenosine monophosphate
**SIN1**	stress-activated protein kinase-interacting protein
**mTORC1/2**	mammalian target of rapamycin complex 1/2
**Adcy10**	Adenylyl cyclase 10
**PKA**	protein kinase A
**CaN**	calcineurin
**CRY2**	Cryptochromes 2

☐ Preliminary screening principles of topological exhaustive method

(1) Output node must be activated by positive regulation from input node or regulatory node;

(2) Regulatory node must be activated by positive regulation from input node or output node;

(3) Either input node or output node must be regulated by regulatory node;

(4) In the topological structures satisfying the aforementioned three conditions, activation between regulatory node and output node must be avoided, but there should be no positive regulatory from input node.

☐ Supplemental Figures

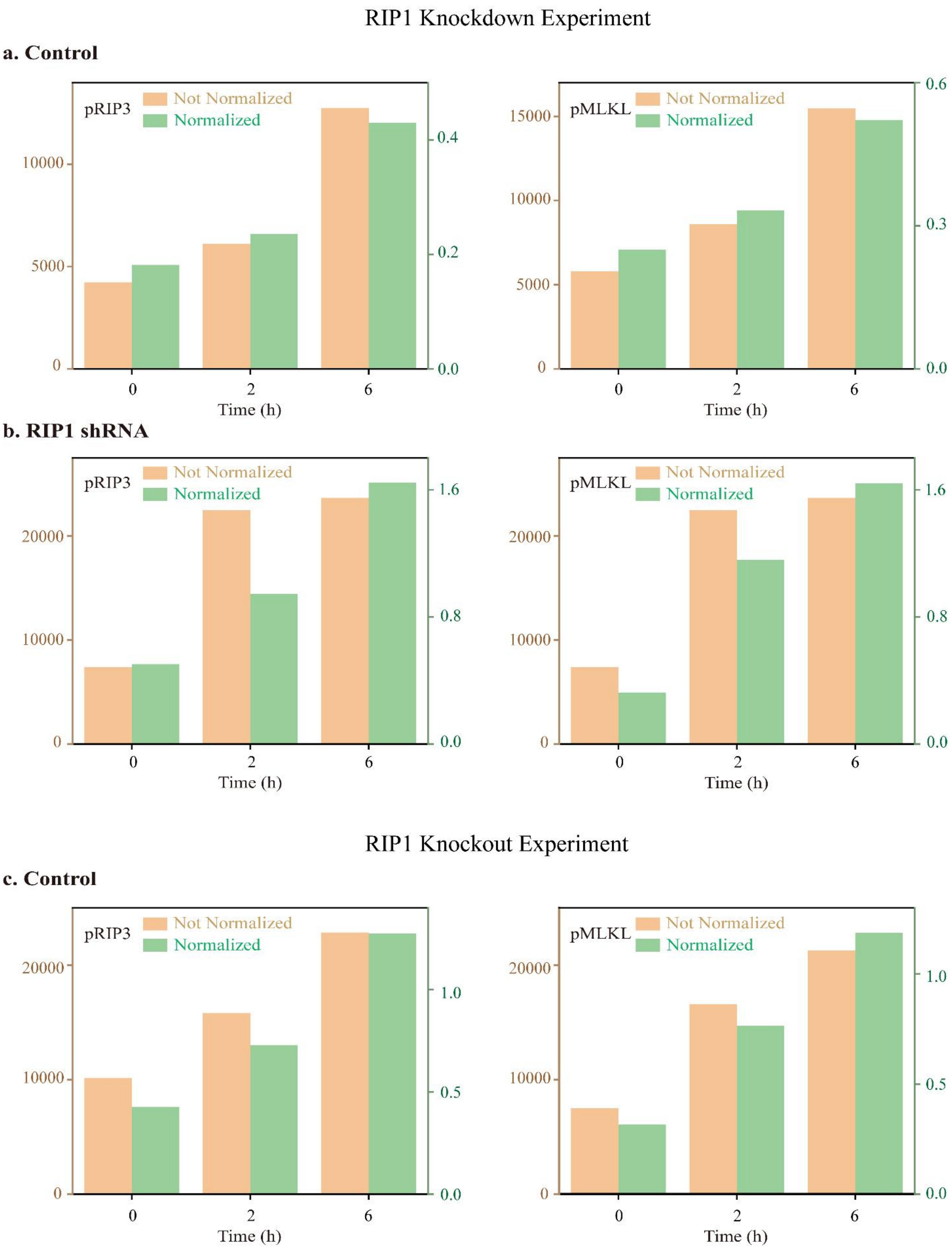

Fig. S1. Quantified western blot results in Fig. 1c of the main text. (a) and (b) show the quantified pRIP3 and pMLKL in control L929 cells and RIP1 knockdown L929 cells. (c) displays the quantified pRIP3 and pMLKL

in control L929 cells of RIP1 knockout experiment. The yellow bars represent the original data of pRIP3 and pMLKL, and the green bars depict the original data normalized to GAPDH.

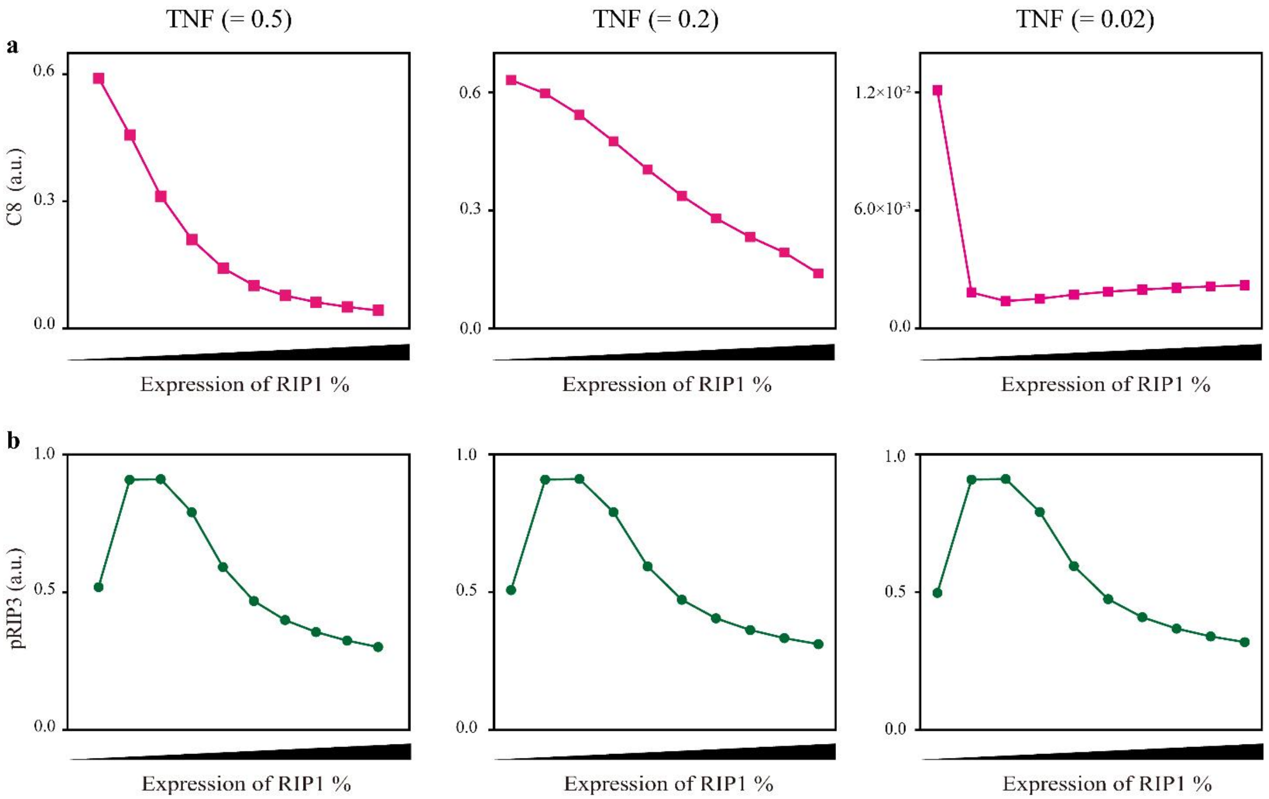

Fig. S2. Effects of different TNF intensities on RIP1 level-dependent activation of caspase 8 (a) and pRIP3 (b).

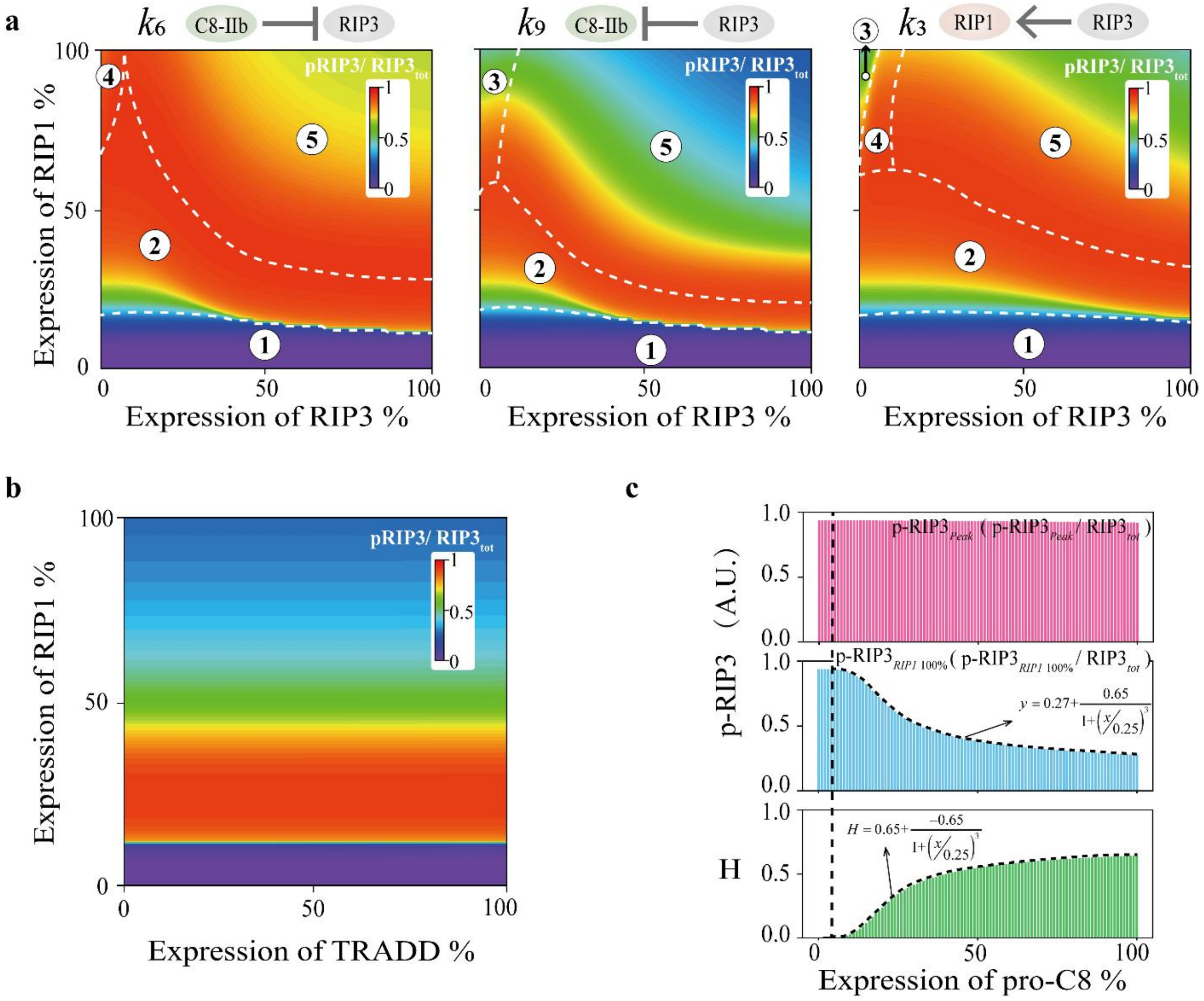

Fig. S3. (a) The relative level of pRIP3 in the RIP3-RIP1 phase space when the terms of k_6 (inhibition of C8 on pRIP3), k_9 (the inhibition of pRIP3 on C8), and k_3 (the positive feedback of pRIP3 on RIP1) are reduced 10-fold, respectively. (b) The relative level of pRIP3 in the TRADD-RIP1 phase space. (c) The variation of pRIP3Peak, pRIP3RIP1_100%, and H with pro-C8 expression level increases.

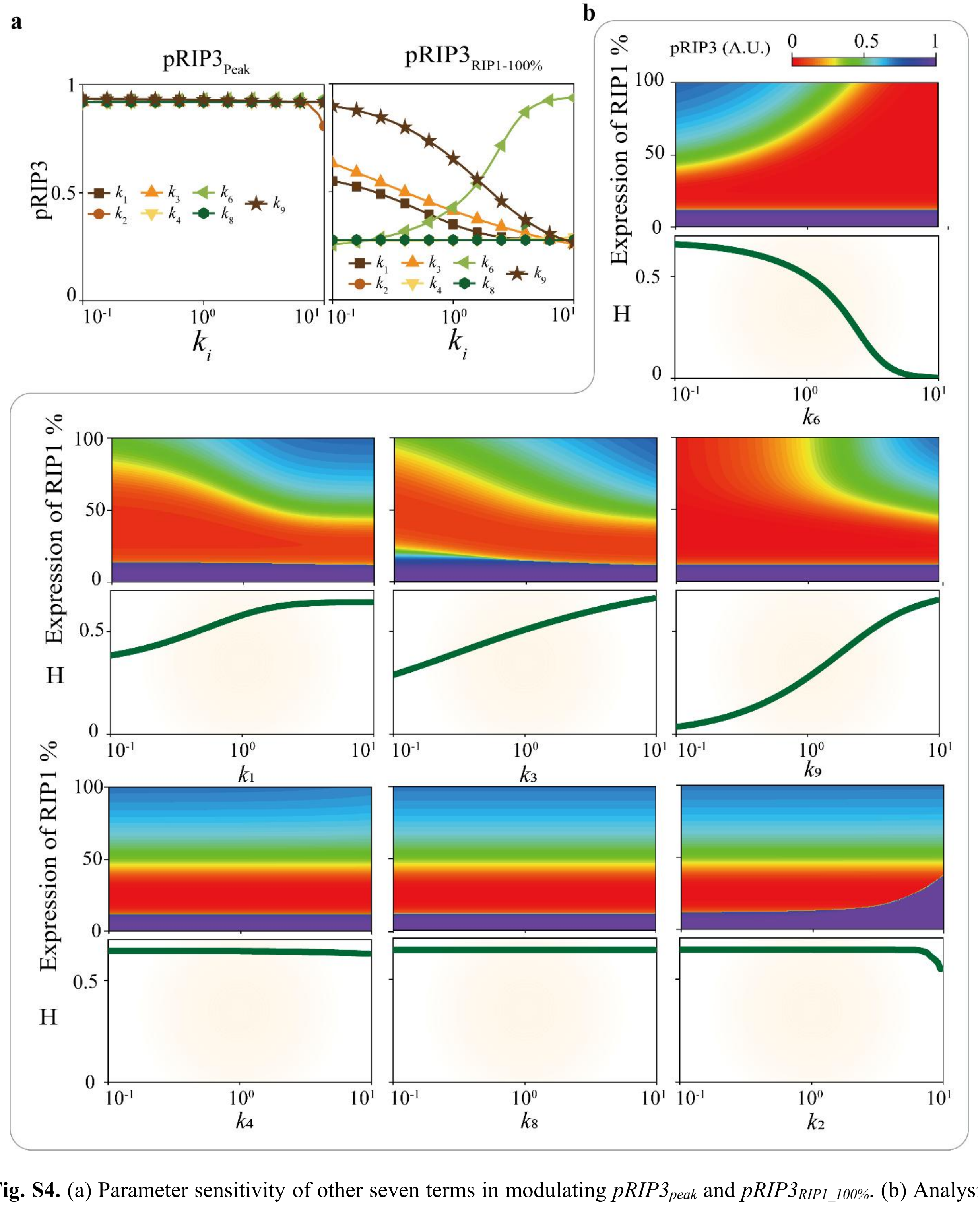

Fig. S4. (a) Parameter sensitivity of other seven terms in modulating $pRIP3_{peak}$ and $pRIP3_{RIP1\_100\%}$. (b) Analysis of the seven terms regulations on pRIP3 and the scale of biphasic dynamics H.

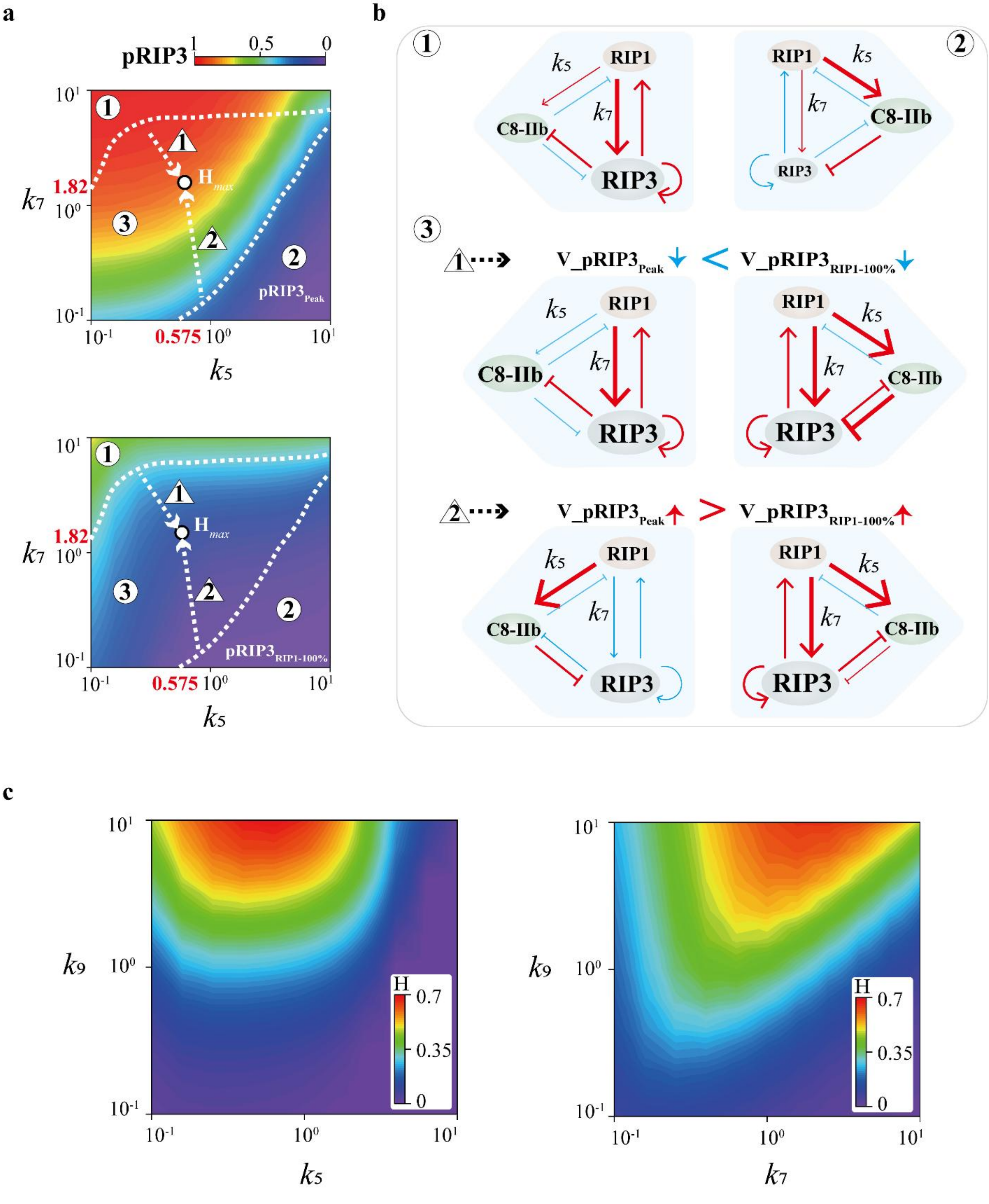

Fig. S5. (a) Levels of $pRIP3_{peak}$ and $pRIP3_{RIP1\_100\%}$ in the k_5-k_7 parameter space, and the phase plane is decomposed into three regions and two processes. (b) Mechanistic analysis of k_5 and k_7 Bell-shaped regulation on pRIP3 biphasic dynamics. In regions 1 and 2, two terms, activation of RIP3 by RIP1 and activation of C8 by RIP1, play the dominant role, respectively. Their corresponding $pRIP3_{peak}$ and $pRIP3_{RIP1\_100\%}$ are both high or both low, resulting in small scales of biphasic dynamics. The decline rate of $pRIP3_{peak}$ in process 1 is lower than

that of $pRIP3_{RIP1\_100\%}$, and the increase rate of $pRIP3_{Peak}$ in process 2 is greater than that of $pRIP3_{RIP1\_100\%}$. (C) Phase diagram of H in k_5-k_7 and k_7-k_9 parameter spaces.

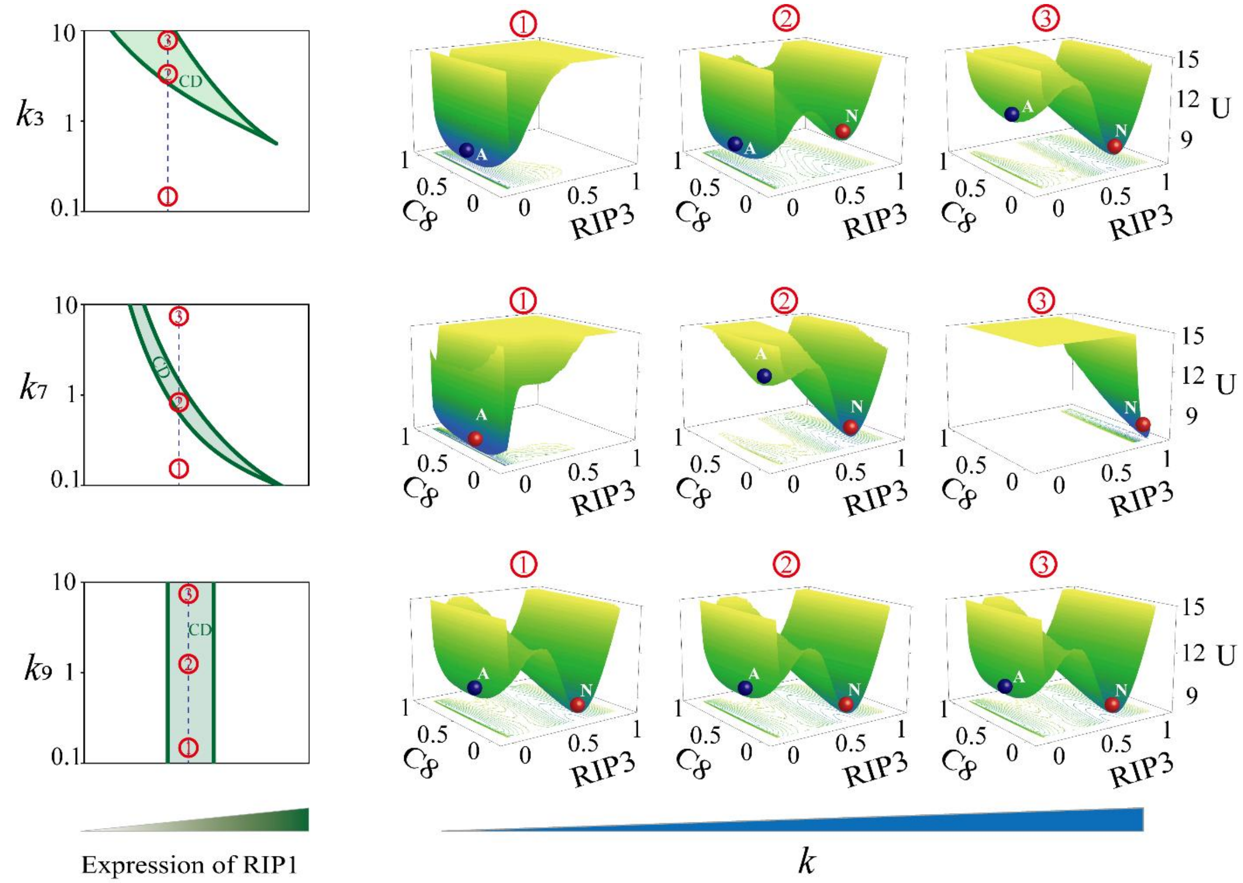

Fig. S6. Phase diagram of the system stability on the co-variation of interaction term (k_3, k_7, and k_9) and the expression of RIP1. The green shaded region indicates the coexistence of apoptosis and necroptosis. The potential energy landscape in C8-RIP3 phase space of the cell death system at three typical values fixed for each parameter.

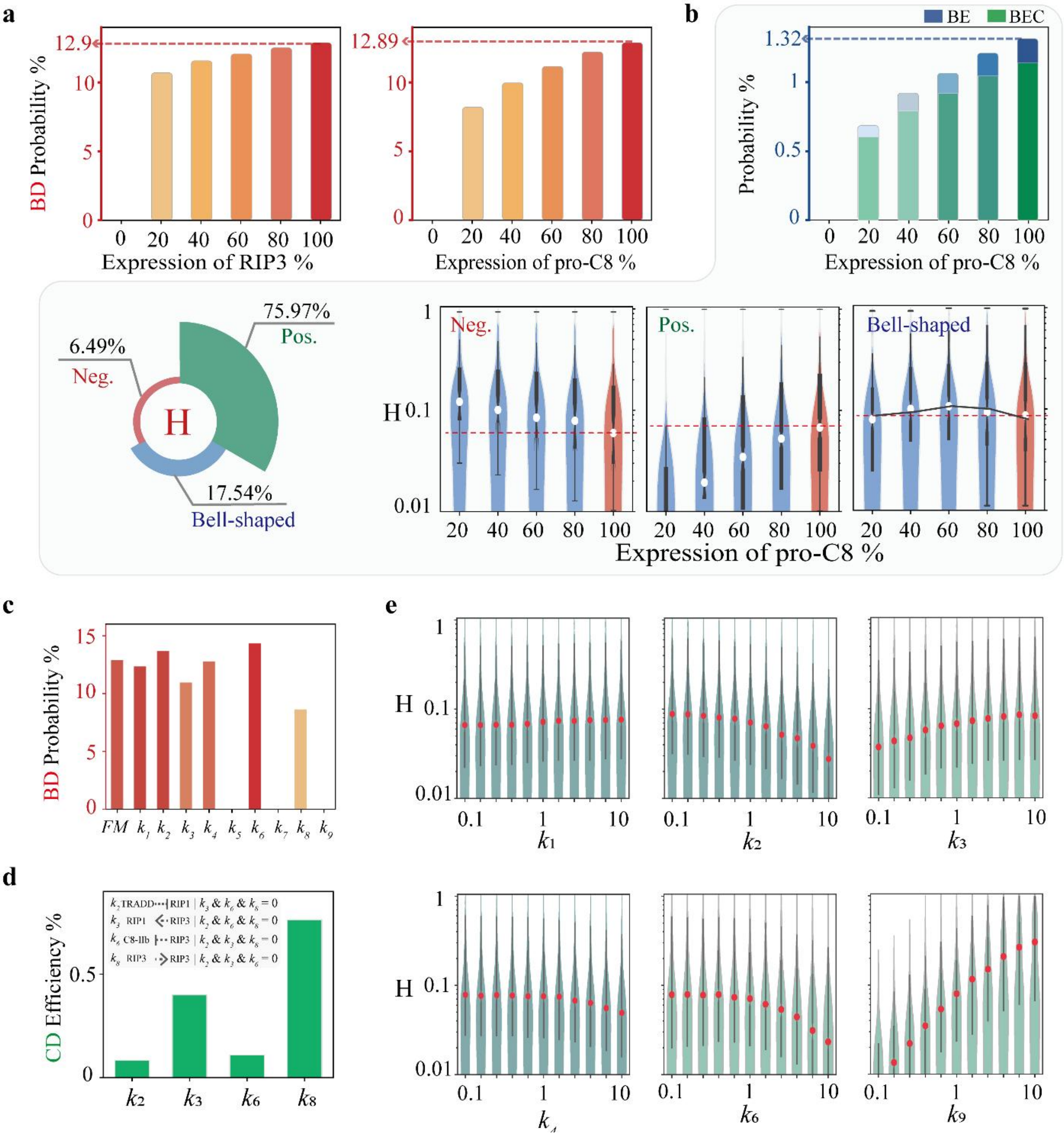

Fig. S7. (a) Random circuit analysis with five representative RIP3 and pro-C8 expression levels to count the probabilities for achieving pRIP3 biphasic dynamics. (b) Random circuit analysis with five representative pro-C8 expression levels to count the probabilities for achieving pRIP3 BE and BEC dynamics, and the statistics of the regulatory behavior of RIP3 on the scale of biphasic dynamics H. (c) The probability of the system achieving biphasic dynamics when all interactions are blocked, respectively. (d) Contribution of all the positive feedback loops in circuit to achieve coexistence dynamics. (e) Statistics of the regulation of other six terms on H.

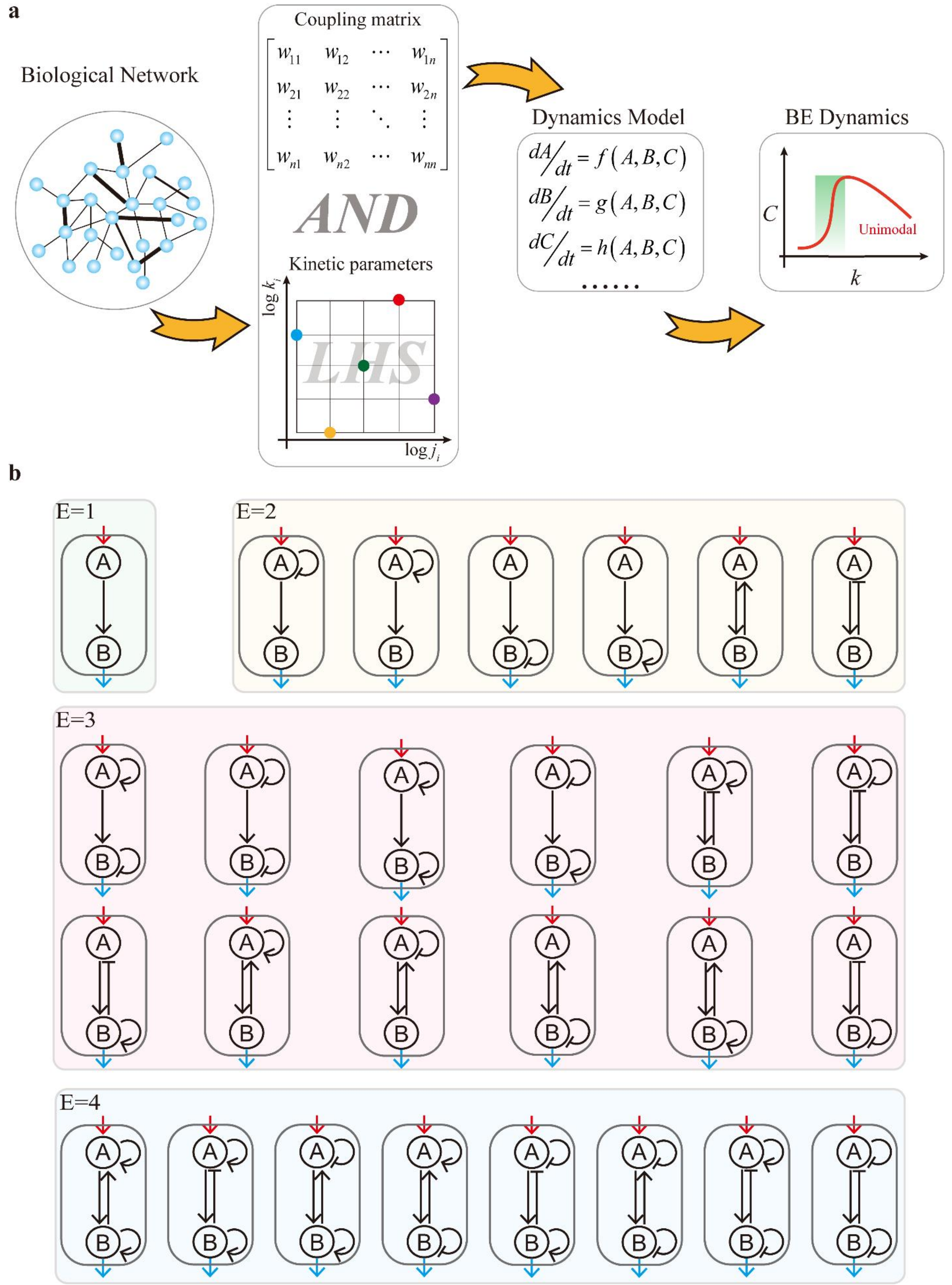

Fig. S8. (a) Workflow for topology-to-function mapping of BE dynamics. (b) The 27 two-node motifs are classified according to the number of terms (connecting edges E).

a

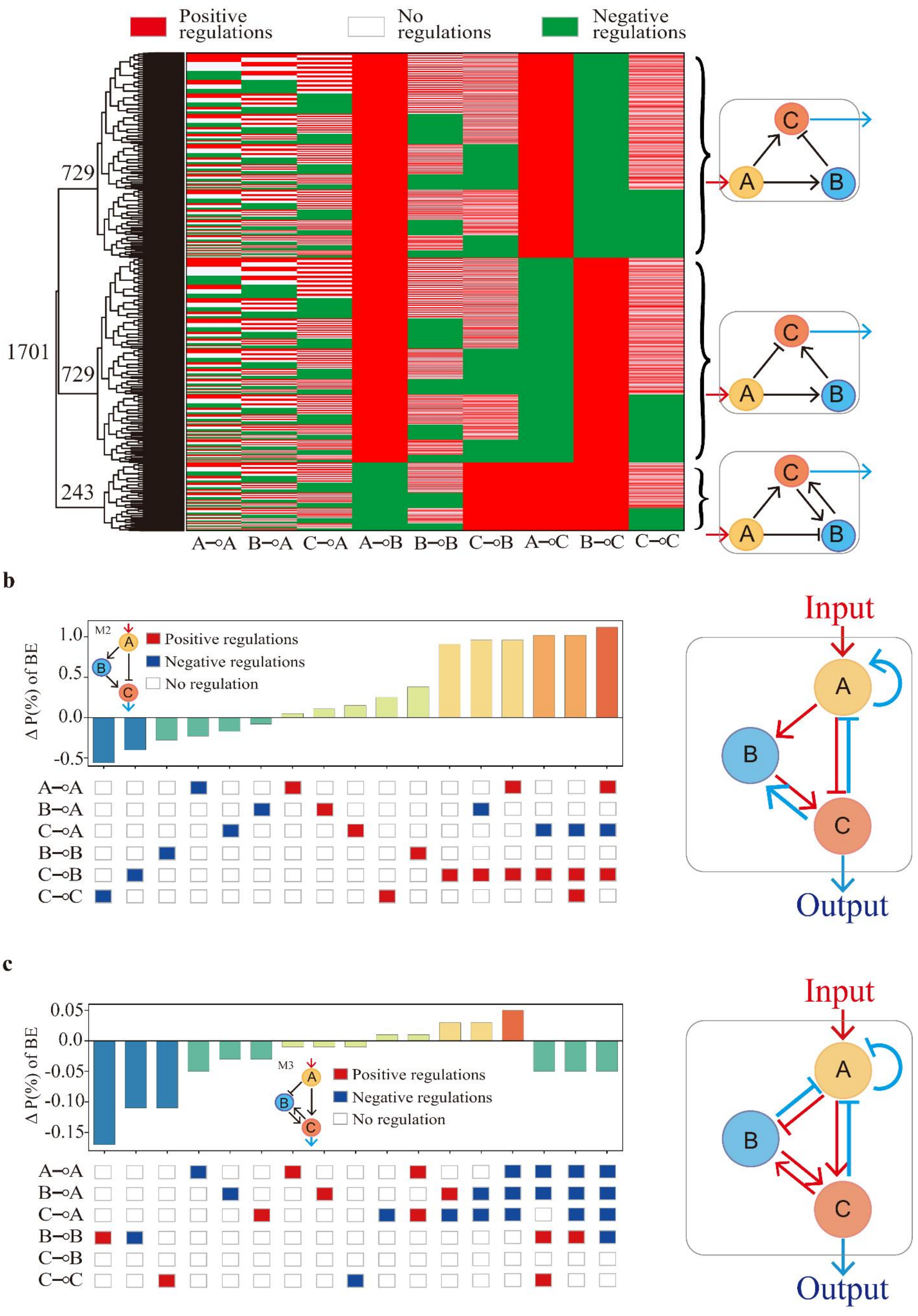

Positive regulations
No regulations
Negative regulations
729
729
243
1701
A⊸A B⊸A C⊸A A⊸B B⊸B C⊸B A⊸C B⊸C C⊸C
b
Δ P(%) of BE
M2
Positive regulations
Negative regulations
No regulation
1.0
0.5
0.0
-0.5
Input
Output
c
0.05
0.0
-0.05
-0.10
-0.15
M3

Fig. S9. (a) Clustering of the 1,701 three-node circuits that can achieve BE dynamics. The core circuits associated with each of the sub-cluster are shown on the right. (b) and (c) Probability statistics of BE dynamics that can be achieved by randomly adding edges based on circuit M2 and M3.

References

[1] X. Li, C.Q. Zhong, R. Wu, et al., RIP1-dependent linear and nonlinear recruitments of caspase-8 and RIP3 respectively to necrosome specify distinct cell death outcomes, Protein & Cell, 12 (2021) 858-876.
